# Dual career in the workplace: co-creation of a conceptual framework by employers and employee-sportspersons incorporating corporate social responsibility and brand alignment

**DOI:** 10.3389/fpsyg.2024.1432850

**Published:** 2024-09-25

**Authors:** Andrea Fusco, Ciaran MacDonncha, Laura Capranica, Chloé Barat, Alberto Bichi, Laurence Blondel, Rosemary Daniel, Mojca Doupona, António José Figueiredo, Ole Keldorf, Giovanni Mattia, Olga Papale, Bratic Milovan, Viktorija Pecnikar Oblak, Valeria Pernetti, Andrej Pisl, Klement Podnar, Lotte Juhl, Ian Sherwin, Nenad Stojiljkovic, Nataša Verk, Giles David Warrington, Michela Mingione

**Affiliations:** ^1^Department of Medicine and Sciences of Aging, G. d'Annunzio University of Chieti and Pescara, Chieti, Italy; ^2^Department of Physical Education and Sport Sciences, University of Limerick, Limerick, Ireland; ^3^Health Research Institute, University of Limerick, Limerick, Ireland; ^4^Department of Movement, Human, and Health Sciences, University of Rome “Foro Italico”, Rome, Italy; ^5^European Athlete as Student (EAS) Network, Asciac, Malta; ^6^European Platform for Sport Innovation (EPSI), Brussels, Belgium; ^7^National Institute of Sport, Expertise, and Performance (INSEP), Paris, France; ^8^Department of Sport Sociology, Faculty of Sport, University of Ljubljana, Ljubljana, Slovenia; ^9^Faculty of Sport Sciences and Physical Education, University of Coimbra, Coimbra, Portugal; ^10^Sciences, Society, and Health, Elite Sport Academy Aarhus (ESAA), Aarhus, Denmark; ^11^Roma Tre University, Rome, Italy; ^12^Department of Human Sciences, Society and Health, University of Cassino and Lazio Meridionale, Cassino, Italy; ^13^Faculty of Sport and Physical Education, University of Niš, Niš, Serbia; ^14^Human Age Institute Foundation, Roma, Italy; ^15^EUSA Institute, Ljubljana, Slovenia; ^16^Department of Marketing Communication and Public Relations, Faculty of Social Sciences, University of Ljubljana, Ljubljana, Slovenia; ^17^University of San Raffaele Roma, Rome, Italy

**Keywords:** concept mapping, dual career networks, value creation, employee wellness, dual career guidelines

## Abstract

**Introduction:**

The purpose of this study was to provide an evidence base and conceptual framework to inform new guidelines for achieving a balance between sports and employment commitments (i.e., dual career, DC) of the employee-sportspersons. To shape a DC discourse in the workplace, the distinct and combined views of the employee-sportspersons (i.e., the Employee), the managers (i.e., the Employer) were considered.

**Methods:**

Following a concept mapping methodology, 257 international participants (25% employers and 75% employee-sportspersons) sorted and rated 50 potential statements associated with DC circumstances and supports in the workplace.

**Results:**

Six distinct clusters emerged, with the combined employers-employee co-creation scenario assigning 6 statements to the micro dimension (Cluster 1 = Workplace Benefits), 4 statements to the meso dimension (Cluster 2 = Role of National Sports Governing Bodies), 19 statements to the macro dimension (Cluster 3 = Dual Career Policy Development), 4 and 5 statements to the organizational dimensions (Cluster 4 = Employee-Employer Collaboration and Responsibility; Cluster 5 = Sport Career Integration), and 12 statements to the policy (Cluster 6 = Workplace Strategies for Dual Career Support) dimension. With respect to the employers, the employee-sportspersons showed higher scores (*p* < 0.05) for importance of clusters 2, 4, and 6, and for feasibility of clusters 2 and 6.

**Discussion:**

These findings suggest priorities for changes within the DC dimensions identified, and envisage flexible models for aligning corporate brand values and corporate social responsibility strategies through meaningful and proactive DC support of the employee-sportspersons in the workplace. The findings provide a rigorously derived evidence base to inform the formulation of new DC workplace guidelines.

## 1 Introduction

In developing the European dimension in sports, one of the priorities of the European Working Plans has been the elite athletes’ right to combine their sport and education/working careers (i.e., dual career, DC) (European Parliament, 2015, 2017, 2020, 2024). To overcome the inter- and intra-country DC policies and provisions resulting from the full competence in sports of the Member States, in 2012 the European Commission published the EU Guidelines on Dual Careers of Athletes, which recommend the promotion of actions in support of the education, vocational training, and employment of athletes (European Commission, 2012). Furthermore, to foster transnational, cross-sectoral, and horizontal cooperation in DC, the European Commission supported European Studies, and European Collaborative Partnerships under the EuRopean Action Scheme for the Mobility of University Students (ERASMUS) + Sport, which aims to exchange best practice and to develop, to transfer, and/or to implement innovative DC practices at European level (Aquilina and Henry, 2010; Capranica and Guidotti, 2016; European Commission, 2016, 2022b; Guidotti et al., 2023). More recently, the European Parliament recognized the DC needs of coaches, physical trainers, referees, and sports managers engaged in structured sport extending the call for DC implementation to all sportspersons (European Parliament, 2021). The efforts of the European Parliament and the European Commission not only contributed to raising the awareness of DC arrangements and stimulating qualitative and quantitative DC research in secondary and tertiary education of athletes in the Member States, but also fostered a worldwide interest, which determined a wide use of the DC term and positioned Europe as a global leader in the holistic development of sportspersons (Guidotti et al., 2015; Condello et al., 2019; Stambulova and Wylleman, 2019; Torregrossa et al., 2021; Vidal-Vilaplana et al., 2022). In fact, the kaleidoscopic European DC discourse has been considered relevant for comparisons with non-EU States (Quinaud et al., 2020; Gjaka et al., 2024).

At present, DC research focused mainly on the barriers, challenges, and opportunities for the combination of sports and secondary and tertiary education of elite athletes to help them in the transition to the labor market at the end of the sports career (Guidotti et al., 2015; Stambulova and Wylleman, 2019; Torregrossa et al., 2021; Vidal-Vilaplana et al., 2022). Furthermore, the IOC offers tailored advice, workshops, learning tools and resources through its Athlete365 Career+ to help elite and Olympic athletes exploring career opportunities for successfully managing the difficult transition from sport to a new career.^1^ In considering that only professional sports and the Military of some Member States could guarantee an economic return to sportspersons for their full-time dedication in sports at national and international levels, the increased duration of sports careers into adulthood urge elite and sub-elite sportspersons to find an employment when their sport salary does not allow them to overcome the financial uncertainty and the economic burden of a prolonged sports career (Allen and Hopkins, 2015; López de Subijana et al., 2020; Barth et al., 2021; McLeod and Nite, 2021; Marshall et al., 2022; Vretaros, 2022). Although sport- and not sport-related companies of the private sector tend to engage elite sportspersons as testimonials for the promotion of their products and for creating meaning and value transfer, they do not consider the European recommendations to offer flexible working conditions and arrangements for enabling sportspersons as employees to train and to compete in athletic events (Seno and Lukas, 2007; Halonen-Knight and Hurmerinta, 2010; Ding et al., 2011; European Commission, 2020a; Moreno et al., 2021; Rai et al., 2021; Mittag et al., 2022; Robnik et al., 2022; Mingione et al., 2024). Sports could contribute to sustainable development through cohesion policies and business-oriented companies should integrate cross-sectorial approaches to promote and strengthen sustainable business opportunities as well as more active lifestyles (European Commission, 2020a). In fact, companies have a social role and obligations to the community, which are achieved through their Corporate Social Responsibility (CSR) strategies, policies, and practices (Yang et al., 2020). In particular, the companies are called to adopt DC policies for ameliorating the working conditions and for facilitating the employment of sportspersons (European Commission, 2012), who could co-create sport-related values to align the company’s internal (i.e., values and vision) and external (i.e., image) dimensions and help leveraging the brands communication of sustainable strategies and practices (Hatch and Majken, 2008; Balmer, 2012; Kumar and Christodoulopoulou, 2014; Mingione, 2015, 2023; Mingione and Leoni, 2020).

Few studies focused on the barriers, challenges, and needs of employee-sportspersons to effectively combine working and sports schedules, in balancing sport/work and family life and relationships, and in supporting themselves and their families (McLeod and Nite, 2021; Moreno et al., 2021; Marshall et al., 2022). The aforementioned lack of research and knowledge was the premise upon which the ERASMUS+ Sport Collaborative Partnership “BRAnd Alignment Value through Dual Career” (BRAVA-DC, 622824-EPP-1-2020-1-IE-SPO-SCP) was funded for 3 years (2021–2023). BRAVA-DC involved seven European nations (Belgium, Denmark, Ireland, Italy, Malta, Serbia and Slovenia) and nine associated partners. The focus of the project was enhancing the European workplace environment so the circumstances and needs of DC employee athletes and coaches can be effectively accommodated while considering the needs and expectations of the employer. The primary aim was by using an evidence-and an eminence-based approach to develop new European guidelines, which specifically support DC in the workplace and facilitate appropriate brand alignment strategies and CSR policies (MacDonncha et al., 2023).

In the context of the BRAVA-DC project, the purpose of the present study was to co-create with both DC employees and employers a list of statements to inform the development of the new workplace DC guidelines and subsequently by using concept mapping software to rank and organize these statements within a new conceptual framework. Concept mapping is a methodology which effectively gathers, integrates and visually and numerically represents the composite thinking of a group of relevant stakeholders around the nature and factor structure and dynamics of a complex social phenomenon, i.e., dual career in the workplace. Concept mapping can for example inform new theory development; intervention, policy and program design and the development of stakeholder informed guidelines to enhance societal processes. Concept mapping involves a methodological framework with a predefined sequence of research phases designed to organize and represent ideas based on the unique integration of qualitative and quantitative methods (Trochim, 1989; Trochim et al., 2008; Falk-Krzesinski et al., 2011; Rosas and Kane, 2012; Varga et al., 2021; Marshall et al., 2022). In brief a range of methodologies (i.e., literature review, focus groups, consensus, etc.) can be used to generate a list of statements/factors relevant to the question of interest. Subsequently the statements/factors are sorted in clusters and rated (i.e., for importance, modifiability) by a sample of relevant stakeholders.

## 2 Methods

### 2.1 Ethical approval and experimental approach to the problem

The BRAVA-DC project obtained ethical approval from the Institutional Review Board at the University of Rome Foro Italico (CAR 84/2021).

To establish a comprehensive European framework for DC at the workplace which represents the experiences, perceptions, opinions and needs of dual career employees and employers from different countries and working environments, the BRAVA-DC project team deemed appropriate to adopt an ethnographic stance (Genzuk, 2003) and a concept mapping process that amalgams real-world insights with scientific expertise in an integrated obligation-opportunity conceptual model (Trochim, 1989; Trochim et al., 2008; Falk-Krzesinski et al., 2011; Rosas and Kane, 2012).

### 2.2 Participant and inclusion/exclusion criteria

Participants represented the dual career (DC) sportsperson, i.e., concurrently involved in sport, and employment and their employers. Employee participants were current and former female and male DC Athletes and Coaches who are or were in paid employment (Public and Private), aged 18 + years and who were involved in sport at high/national/international levels. Employer participants were private sector male and female corporate or sport managers (i.e., corporate marketing (CM) managers, CSR managers, human resources (HR) managers, any high/mid-level manager) in small (10–49 employees), medium (50–249) and large (250+) size companies.

Participants were recruited through the strategic utilization of the personal networks and confidential databases of the BRAVA-DC project team members. Participation was voluntary and based on an informed consent which ensured confidentiality of the data. Operationally, study participants were divided into three cohorts (i.e., DC Employers, DC Employees and combined), which allowed for a comprehensive exploration of the views of each cohort and their combined views.

### 2.3 Concept mapping

The concept mapping procedure encompassed four stages, bridging in-person and web-based interactions to provide a well-rounded exploration of perspectives and to enhance the robustness of the framework:

1. Initial preparation: To ensure a shared understanding, the BRAVA-DC project team established guidelines encompassing common terminology, and standard operating protocols for gathering evidence-based (i.e., literature review) and eminence-based (i.e., in-depth interviews, national focus groups, consensus and validation by DC experts, face to face interactions, and web-based surveys) knowledge, and inclusion criteria for participants.
2. Generation and validation of statements: The conceptual framework was informed by a comprehensive list of statements. A preliminary list of statements was initially informed by a range of evidence- and eminence-based methodologies, i.e., the outcomes of the scientific literature (Mingione et al., 2024), in-depth interviews and national focus groups. The preliminary list was then further refined and validated/approved by the consensus opinion of the BRAVA-DC project team and by DC experts.
3. Structural organization (sorting and rating): To structure the conceptual framework, employee-sportspersons and employers engaged in the sorting and rating of the statements.
4. Data analysis and interpretation: The final phase involved both a quantitative and qualitative analysis of the generated sorting and rating data. Specifically, the analysis synthesized the viewpoints of DC Employees and Employers into a conceptual framework reflecting the complexity of DC in the workplace.

Thus, the development of the BRAVA-DC framework was a collaborative effort, with active engagement from the BRAVA-DC project team and a diverse panel of stakeholders representing the DC employees, corporate employers, sports managers and academics from across Europe. This inclusive and comprehensive approach not only validates the framework developed but also ensures its relevance and applicability in diverse cultural and organizational contexts.

### 2.4 Concept mapping

The BRAVA-DC project team implemented standard operating protocols to ensure consistency in all data collection methodologies. The implementation of this phase involved a rigorous research design to achieve the project aim (new workplace DC guidelines) which encompassed tailored methodologies and analyses and considered differences between countries, sports, and work environments influenced by various social and cultural contexts (Genzuk, 2003). The key methodologies utilized to generate a preliminary list of statements to inform the formulation of the new European workplace DC guidelines were review of extant literature, in-depth interviews and focus groups. According to the literature Tracy (2010) and Smith and McGannon (2018), the qualitative standard of the BRAVA-DC research was based on (1) worthiness of the topic; (2) rigor regarding the coherence of the research questions, the recruitment of stakeholders, and the data collection and synthesis; (3) transparency of the research methods; (4) credibility in fostering different perspectives of the involved stakeholders; (5) resonance in raising the awareness of DC at the workplace.

Initially, to collect evidence-based knowledge on factors influencing the DC of employee-sportspersons, a systematic literature review was organized following a consensus on the search strategy for electronic databases and a snowballing technique to overcome limitations of the electronic search, inclusion criteria for the research topics, and the methodology for data extraction and analysis (Mingione et al., 2024). In considering the novelty of DC in the workplace, the aforementioned eminence-base knowledge also encompassed in-depth interviews with composite target groups of employee-athletes/coaches. The in-depth interviews considered the following aspects: purpose and context and relevant definitions; background and nature of work and sport; concept and understanding of DC; balancing work-sport careers and work-life balance; existing workplace DC support and responsibilities; workplace brand value alignment with DC support; DC barriers/challenges and expectations regarding the workplace support. Furthermore, five national focus groups were organized in the Member States of the BRAVA-DC Partners (i.e., Belgium, Denmark, Ireland, Italy, and Slovenia) and involved participants as previously outlined.

The focus groups provided a wealth of insights to generate the preliminary list of statements (Kamberelis and Dimitriadis, 2005; Parker and Tritter, 2006). The following questions were discussed within the focus groups: (1) describe how the work-sport careers of employee-athletes/employee-coaches are supported in your workplace - what are the characteristics of this support; (2) what are the key challenges for the dual career (Sport and Work) employee; (3) how best can the work-sport career of employee-athletes/employee-coaches be supported by the workplace – what does best practice look like; (4) describe the alignment in your workplace between the support for DC and the corporate brand/CSR polices that are promoted – what are the characteristics of the alignment and (5) how best can the workplace align the corporate brand/CSR policy with sport and DC.

Individual thoughts and experiences were stimulated in a non-judgmental manner, fostering exchanges and eliciting feedback on each other’s perspectives.

### 2.5 Generation and validation of statements to inform the new DC in the workplace guidelines

As outlined above three key methodologies informed the preliminary list of statements. To generate a final list of potentially relevant statements crucial to the development of the DC in the workplace, informed consent was provided by 22 employee-sportspersons (Age: 38.4 ± 10.4 years; F: 50%; Athletes/Former Athletes: 59%; Coaches: 36%; Referees: 5%) involved in the in-depth interviews and 59 participants (Age: 31–60 years; F: 36%; 54% employers; 46% employee-sportspersons) in the national focus groups.

The data from the review of the extant literature and the in-depth interview methodologies was examined by members of the project team (*n* = 4) and via consensus 19 unique factors were identified and concisely articulated for consideration (i.e., “support/valorize the mutual benefits of complementarity between sports and work”). The key factors identified from each of the five national focus groups for each of the five questions posed were concisely articulated and organized on an excel sheet (i.e., “No actual formal support policy in place”). In support of this process, each focus group provided an initial report, which included group agreed-upon statements, based on the five questions posed. This extensive data was examined by members of the project team (*n* = 5); for each of the five question posed common and unique responses were identified and revised statements were articulated – the interpretation of the original focus group responses into a revised statement was confirmed via consensus. This process resulted in a substantial reduction and refinement of the original data stemming from the focus groups. The next stage of revision involved using the following anchor question to articulate statements which more effectively represented the views of all focus group participants: “Please rank the importance of the following statements to inform and/or for inclusion in new European guidelines for workplace support for the DC employee Athlete/Coach and appropriate workplace brand alignment strategies and CSR policies.” In total a preliminary list of 85 statements was developed based on the data from all methodologies used, i.e., “To accept that employers should not have lower expectations/standards of work because of DC commitment - accommodation of DC is what is required.” It is important to note that the flavor of all responses from the focus group participants were incorporated into the preliminary list – this interpretation was once again confirmed via consensus. A final stage of consensus and refinement was then subsequently implemented where upon each preliminary statement was considered using the following criteria: (1) same or similar statement noted; (2) statement can be combined with other; (3) statement should be fragmented; (4) statement unclear. The outcome of this consensus step resulted in a final list of 50 unique statements. A further step of validation/approval involved online feedback in July 2022 from 37 DC experts (F: *n* = 13, 35%; M: *n* = 24, 65%) from 16 countries (Belgium, Brazil, Croatia, Denmark, Germany, Iceland, Ireland, Italy, Latvia, Lithuania, Poland, Portugal, Romania, Slovenia, Spain, Sweden). The experts were known to the project team and drawn from existing databases. Participants were asked to rate the clarity of the statements by means of a 5-point Likert scale (from 1 = Not clear at all, to 5 = Very clear) and to suggest any additional statements to be included. Overall, mean clarity was 4.2 ± 0.2. Seven statements did not meet the 4-point threshold for clarity (range: 3.6–3.8) and were subsequently revised. The final set of 50 statements developed through rigorous methodologies and an exhaustive consensus and expert approval process were now available for the online concept mapping exercise.

### 2.6 Structural organization (sorting and rating)

The GroupWisdom™ online concept mapping platform^2^ was used to facilitate the participants’ concept mapping tasks, which encompass the assessment of the importance and feasibility to implement of the 50 statements by means of a 5-point Likert scale (from 1 = Not at all important/feasible, to 5 = Very important/feasible), and the sorting of all statements into clusters. To identify any potential technical or interpretation issues, the BRAVA-DC project team engaged in pilot trials to verify that efficient and error-free data collection was effectively and correctly occurring.

A confidential questionnaire collected a range of participant details, including gender, nationality, workplace size (i.e., number of employers), age (from 21 to 61+ years), primary sports role and competitive level of sportspersons (i.e., national, international, amateur, professional), primary working role (i.e., employer, employee) and sector (i.e., private, public). Regardless of their involvement in the previous phases of the project, potential participants for the concept mapping exercise were identified from the BRAVA-DC national databases, with a target of 300 potential participants established across the seven national partners. All potential participants received a pre-notification email outlining the BRAVA-DC project’s aim to develop a European framework for DC support in the workplace, based on an integrated obligation-opportunity conceptual model. It also included information on the time required to complete and the nature of the concept mapping exercise. Participants were explicitly informed of the voluntary nature of their involvement and their right to withdraw at any point without justification and were assured the of the confidentiality for all responses. Consent was considered implied upon survey submission. Upon response to the pre-notification email, the potential participants received a second email providing comprehensive instructions to utilize the online concept mapping platform, a unique participant code to allow the anonymous linkage between the background questionnaire and the concept mapping responses, a link to the background questionnaire, and a personalized link for platform access to engage in and complete the concept mapping exercise. Participants were offered the option to complete the task either online at home or during a face-to-face meeting, with assurance that responses would remain anonymous and confidential. The detailed instructions provided: (a) the list of the 50 statements, and (b) clear instructions of the two tasks to be completed. Task 1 - Rating the importance and feasibility of each statement following the instructions: “Please rate the importance of the following 50 statements in relation to the development of new European guidelines for DC supports, strategies and policies in the workplace. Please rate each statement on a scale of 1 to 5 (from 1 = Not at all important, to 5 = Very important)” and “Please rate the feasibility to implement the characteristics of each of the following 50 statements in the workplace of the DC employee and DC employer. Please rate each statement on a scale of 1 to 5 (from 1 = Not at all feasible, to 5 = Very feasible).” Task 2 – Based on personal logic and reasoning organize the statement into a maximum of 10 clusters. The following instruction was provided: “To complete the sorting activity, you must first search for similarities across the statements and subsequently organize them into smaller groups (maximum 10 groups) which are logical and make sense for you.”

Participants were encouraged to review and finalize their decisions before submission. In understanding the potential impact of longer surveys on response rates, participants were also recommended to complete the tasks in two sessions with two reminder emails sent to encourage participation, and several reminder emails to invite the respondents to finalize both the ratings and sorting tasks.

### 2.7 Data analysis and interpretation

Before starting the data analysis, one of the co-authors with a certified expertise in Concept Mapping procedure, reviewed the participants’ sorting and rating responses to confirm their adherence to the provided guidelines. Descriptive statistics including mean, standard deviation, and frequency of occurrence were calculated for both demographic data and statement rankings. Subsequently, the GroupWisdom™ online platform was utilized for data analysis, employing a square symmetric similarity matrix generated from the sorted statements. A two-dimensional non-metric multidimensional scaling technique was applied, mapping each statement as a point on an x-y spatial “point map” for each cohort. Hierarchical cluster analysis was utilized to cluster the points representing statements on the point maps. For the employer, employee, and both cohorts, during three meetings the BRAVA-DC project team systematically assessed various cluster solutions following the recommended steps by Kane and Trochim (2007) with the objective to collapse clusters logically for emphasizing distinct themes among the statements. This process represented a mixed method approach to the data analysis blending the quantitative statistical outcomes with a qualitative interpretation by the project team. Then, clusters were named based on group names provided by participants and the consensus of the project team.

Once the cluster maps agreed and finalized, Go-Zones were established, showcasing statement ratings on importance and feasibility to implement in relation to mean ratings. The top right quadrant (green - IV) of the Go-Zone comprised statements considered both more important and more feasible than the average. Pearson Product Moment Correlation coefficient was used to highlight a correlation between “Importance” and “Feasibility” for both employer and employee cohorts. The validity of the sample sizes was verified following the study and guidelines outlined in accepted research designs, indicating that the sample sizes achieved align with established standards for this type of research (Trochim, 1989; Rosas and Kane, 2012).

*T*-tests were employed to calculate the *t*-value, degrees of freedom, and significance level (*p*-value 0.05) for comparing the Employers and Employees cohorts within the Co-Creation Scenario (combined employee and employer data) on similar ratings (Importance and Feasibility) for each cluster. Following this analysis, Pattern Matches cluster comparison graphs were generated to visualize and compare the average importance and feasibility ratings for each of the employee and employer clusters matched against in the co-creation cluster. This graphical representation aids in discerning the differences and similarities between the two cohorts. The Pattern Match tool employs a ladder graph layout, utilizing statement averages from approved rating data to derive cluster averages. Each vertical axis on the graph shows a distinct variable, and cluster positioning is based on their respective rating values. Connecting lines link the same cluster on both sides of the graph, maintaining consistency with the colors established during the cluster rating map editing.

Cohen’s d effect sizes (ES) were calculated to assess the magnitude of differences between cohorts, accounting for unequal sample sizes, for each cluster and rating comparison. As the study involved cohorts with unequal sample sizes, the formula for calculating pooled standard deviation was adjusted accordingly. Then, Cohen’s d was obtained by dividing the mean difference between groups by the pooled standard deviation. The derived ES were interpreted following Cohen’s benchmarks (i.e., approximately 0.2 indicating a small effect, around 0.5 a medium effect, and approximately 0.8 or higher suggesting a large effect). All calculations were performed after conducting *t*-tests to evaluate group differences. STATA 18 (StataCorp LP, College Station, TX, United States) was used for all the statistical inferences and statistical significance was set at *p* < 0.05.

## 3 Results

### 3.1 Characteristics of the sample

A total of 257 participants took part in the concept mapping exercise, with 25% representing Employers and 75% Employees, at National and International level. The gender distribution was 35% Females and 65% Males. Participants represented 22 countries (Slovenia: 28%; Serbia: 20%; Italy: 18%; Ireland: 11%; Denmark: 7%; Croatia: 4%; France and Slovakia: 2%; Bosnia-Herzegovina Ghana, Spain, Portugal, Lithuania, Poland, Finland, Germany, Greece, United Kingdom, Romania, Latvia, India, Iran: <2%). Participants ranged in age from 21 to 61+ years, with the highest frequency of occurrence belonging to the 21–30 years (43%) and 31–40 years (22%) age groups. The workplace size exhibited an even distribution across very small (26%), small (24%), medium (25%), and large (25%) scales. Regarding the sport and managerial distributions, 44% were athletes (actual and former), 26% coaches (Head Coach, Assistant Coach, and Physical/Performance Coach), 7% CSR managers, 6% CM managers, 17% self-entrepreneurs or self-employed, respectively.

#### 3.1.1 Employers

**Figure 1:**
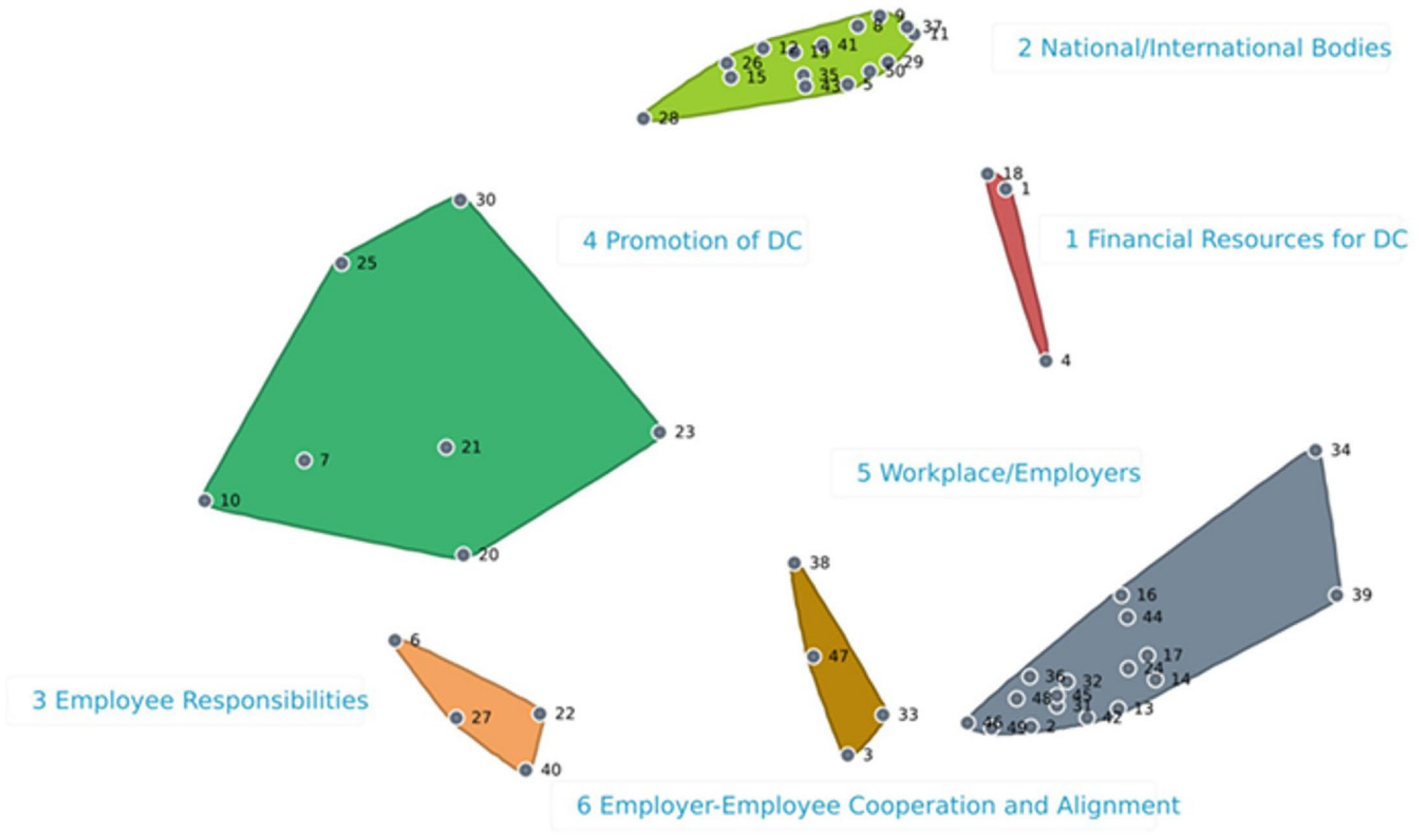
Employers cluster map. *DC, Dual career; *Colored clusters: Red = 1. Financial re-sources for dual career; Light green = 2. National/international bodies; Orange = 3. Employee responsibilities; Dark Green = 4. Promotion of dual career; Grey = 5. Workplace/employers; Brown = 6. Employer-employee cooperation and alignment. *The number represents the exact statement.

**Figure 2:**
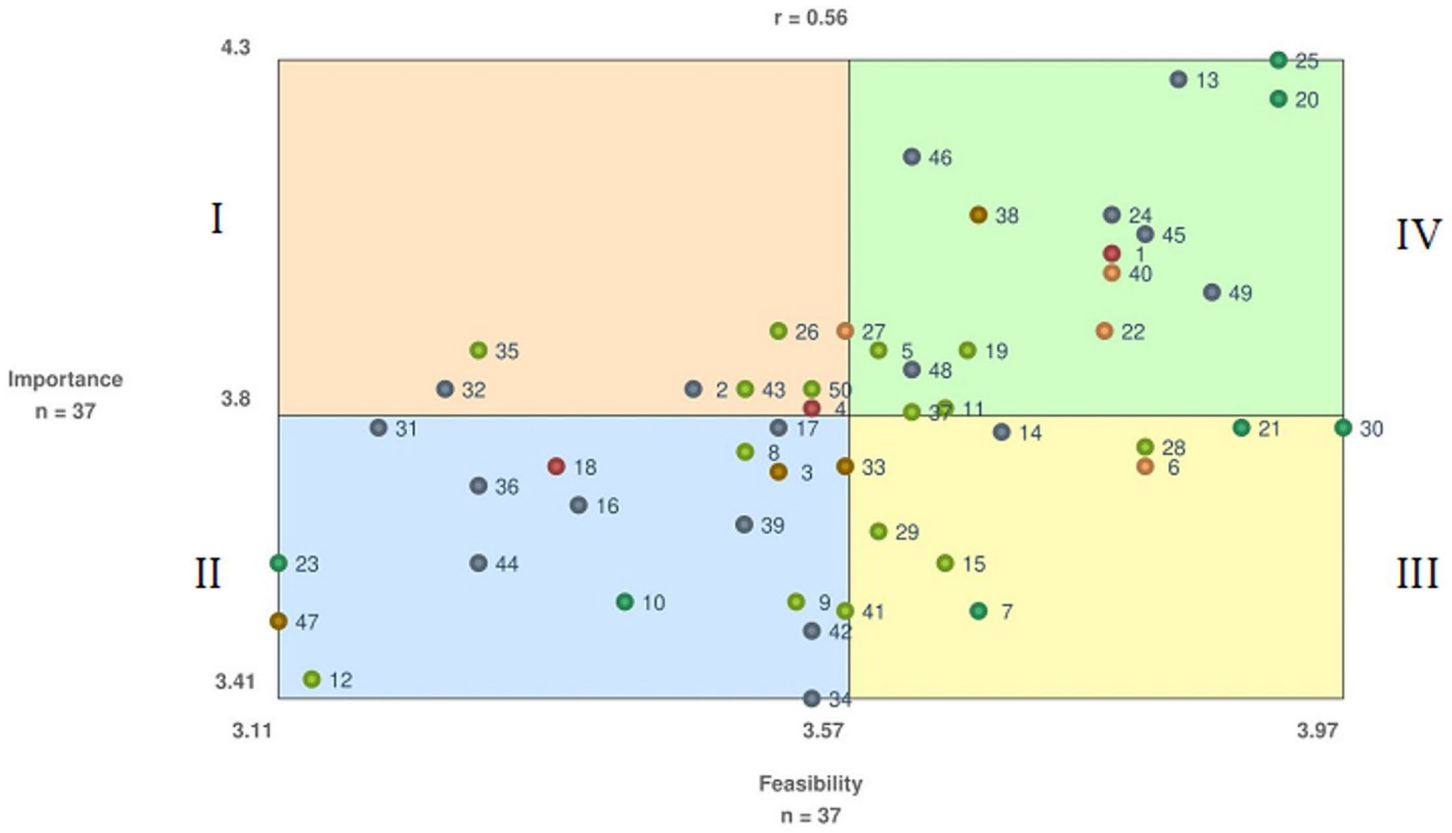
Employers Go-Zone graph. *Legend Go-Zone quadrants: I -Orange, top left = Low importance/high feasibility; II - Blue, bottom left = Low importance/low feasibility; III - Yellow, bottom right = Low importance/high feasibility; IV - Green, top right = High importance/high feasibility. *Colored dots represent clusters: Red = Financial resources for dual career; Light green = National/international bodies; Orange = Employee responsibilities; Dark green = Promotion of dual career; Grey = Workplace/employers; Brown = Employer-employee cooperation and alignment. *The number represents the exact statement.

The 29 Employers sorted the 50 statements in 3 to 10 groups, with an average of 5 groups. The BRAVA-DC research team identified a six-cluster solution (Figure 1) as the best fitting and coherent, having a 0.17 stress value within the accepted range for a good fit between the 3D data and the 2D map (Rosas and Kane, 2012). Furthermore, the cluster map demonstrates a spatial relationship similarity between statements, where closer proximity indicates a higher frequency of statements grouped by the participants. According to the literature (Capranica and Guidotti, 2016), one cluster (Employee Responsibilities, 4 statements) related to the micro (i.e., the employee) DC dimension, one cluster (Employer-Employee Cooperation and Alignment, 4 statements) related to the meso (i.e., interpersonal relationships) DC dimension, one cluster (Workplace/Employers, 17 statements) related to the macro (i.e., working environment) DC dimension, two clusters related to the organizational (Financial Resources for Dual Career, 3 statements; and Promotion of Dual Career, 7 statements) DC dimension, and one cluster (National/International Bodies, 15 statements) related to the policy dimension, respectively. A total of 37 Employers rated the importance and feasibility of the 50 statements. Supplementary Table 1 show mean importance and feasibility ratings within each cluster for each statement and relative go-zone graph quadrant. For importance, the overall rating was 3.8 ± 0.2-point (range: 4.3–3.4-point), with the cluster Employee Responsibilities showing the highest mean value and the cluster National/International Bodies the lowest one. For feasibility, the overall rating was 3.6 ± 0.2-point (range: 4.0–3.1-point), with the highest value emerging for the cluster Employee Responsibilities and the lowest value for the clusters Workplace/Employers, Employer-Employee Cooperation and Alignment, and National/International Bodies. Figure 2 illustrates the Go-Zone, with an *r* = 0.56 indicating a predictable alignment between the x and y variables. In the top right quadrant (IV) the 16 statements the Employers considered most important and feasible are reported, with the statements 25, 13, and 20 showing the highest values. Conversely, 18 statements with values below the mean importance and feasibility are included in quadrant II, with statements 12, 47, and 23 being considered less feasible.

#### 3.1.2 Employees

**Figure 3:**
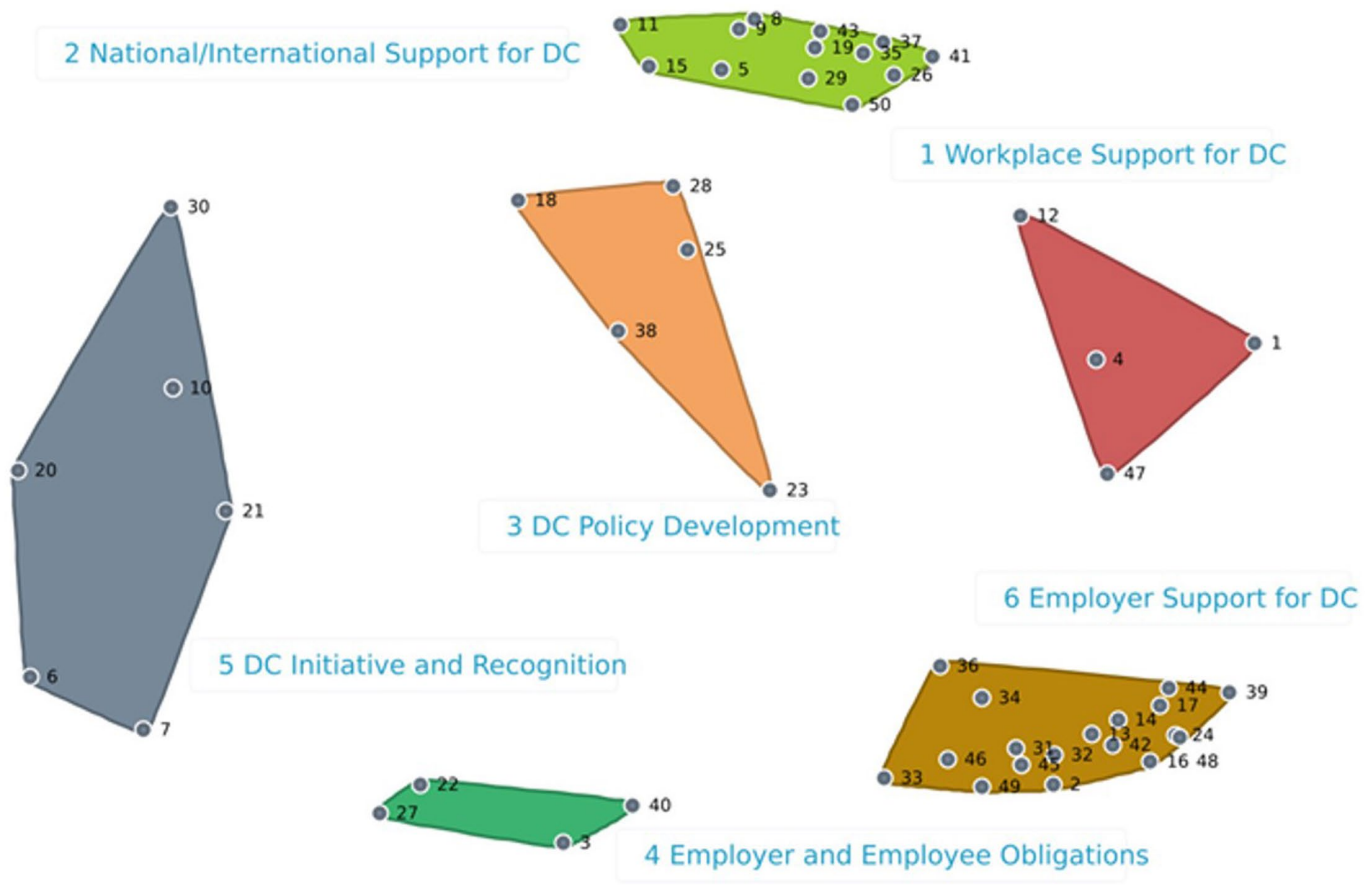
Employees cluster map. *DC, Dual career; *Colored clusters: Red = 1. Workplace support for dual careers; Light green = 2. National/international support for dual career; Orange = 3. Dual career policy development; Dark green = 4. Employer and employee obligations; Grey = 5. Dual career initiative and recognition; Brown = 6. Employer support for dual careers. *The number represents the exact statement.

**Figure 4:**
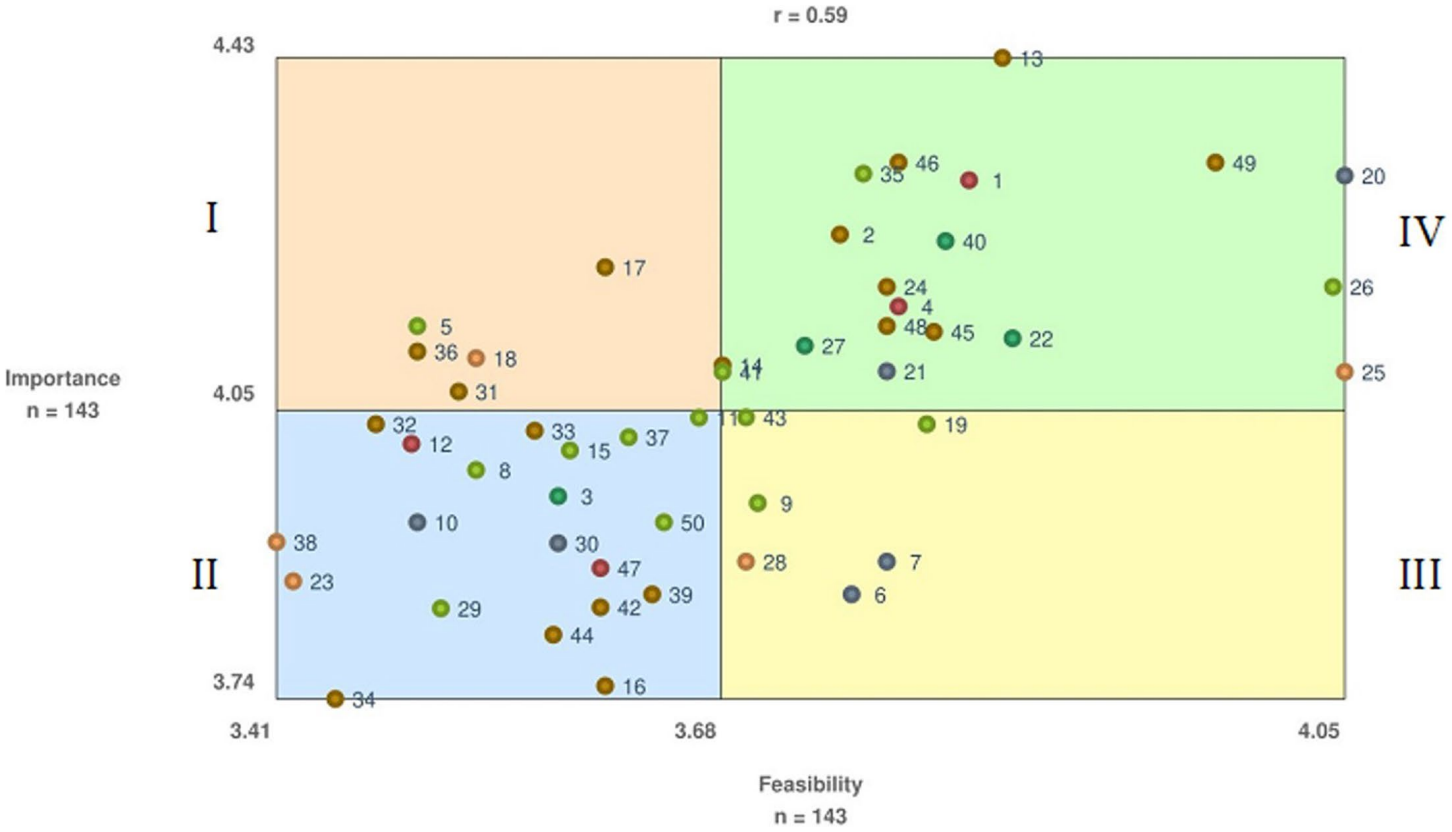
Employees Go-Zone graph; *Legend Go-Zone quadrants: Green, top right = High importance/high feasibility; Yellow, bottom right = Low importance/high feasibility; Orange, top left = Low importance/high feasibility; Blue, bottom left = Low importance/low feasibility. *Colored dots represent clusters: Red = Workplace support for dual careers; Light green = National/International support for dual career; Orange = Dual career policy development; Dark Green = Employer and employee obligations; Grey = Dual career initiative and recognition; Brown = Employer support for dual careers. *The number represents the exact statement.

A total of 87 Employees sorted the 50 statements in 3–10 groups, with an average of 5 groups. For the Employee too, the BRAVA-DC research team identified a six-cluster solution, which showed a 0.15 stress value (Figure 3). The analysis shows that one cluster (Dual Career Initiative and Recognition, 6 statements) related to the micro (i.e., the employee) DC dimension, one cluster (Employer and Employee Obligations, 4 statements) related to the meso (i.e., interpersonal relationships) DC dimension, one cluster (Employer Support for Dual Careers, 18 statements) related to the macro (i.e., working environment) DC dimension, two clusters related to the organizational (Workplace Support for Dual Careers, 4 statements; and Dual Career Policy Development, 5 statements) DC dimension, and one cluster (National/International Support for Dual Career, 13 statements) related to the policy dimension, respectively. With respect to their Employers counterparts, 143 Employees attributed higher scores for importance (4.0 ± 0.2-point; range: 4.4–3.7-point) and feasibility (3.7 ± 0.2-point; range: 4.1–3.4-point) (Supplementary Table 2). For importance, the majority of the clusters showed 4.0-point mean values, with the clusters number 1 and 6 showing the highest value (4.1-point). For feasibility, mean values ranged between 3.7-point (clusters number 1, 2, 4, and 5) and 3.6-point (clusters 3 and 6). Figure 4 illustrates the Go-Zone, which reported a *r* = 0.59, indicating a predictable alignment between the x and y variables. In the top right quadrant (IV) are reported the 19 statements the Employees considered most important and feasible, with the statement 13 showing the highest values for importance and the statements 20, 25 and 26. The quadrant II included 20 statements, with the statement 23 and 38 showing the lowest value for feasibility and the statement 34 the lowest value for importance.

#### 3.1.3 Employers/employees co-creation

**Figure 5:**
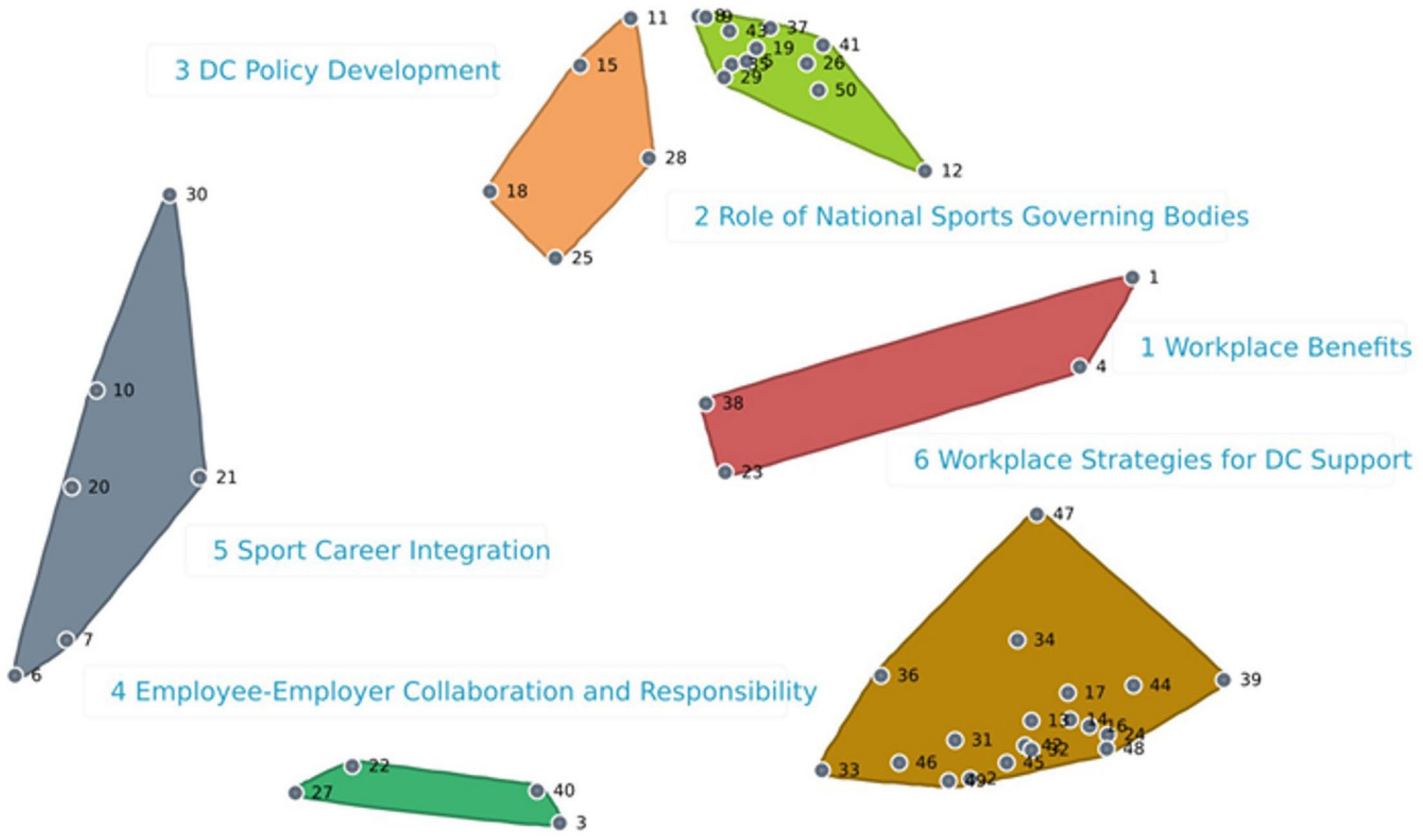
Employers-employees co-creation cluster map. *DC, Dual career; *Colored clusters: Red = 1. Workplace benefits; Light green = 2. Role of national sports governing bodies; Orange = 3. Dual career policy development; Dark green = 4. Employee-employer collaboration and responsibility; Grey = 5. Sport career integration; Brown = 6. Workplace strategies for dual career support. *The number represents the exact statement.

**Figure 6:**
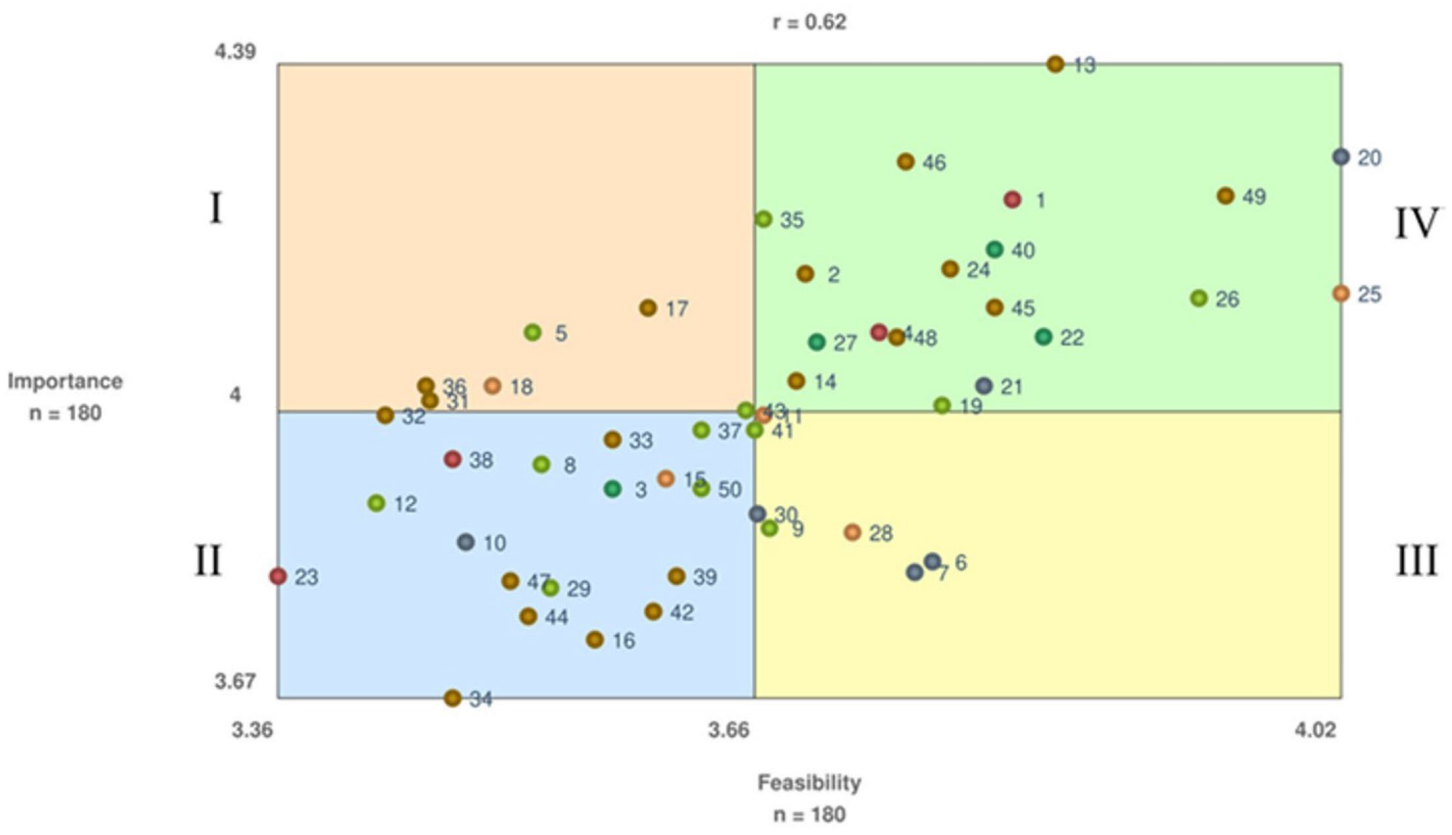
Employers-employees co-creation Go-Zone graph; *Colored dots represents clusters: Red = Workplace benefits; Light Green = Role of national sports governing bodies; Orange = Dual career policy development; Dark green = Employee-employer collaboration and responsibility; Grey = Sport career integration; brown = Workplace strategies for dual career support. *The number represents the exact statement.

**Figure 7:**
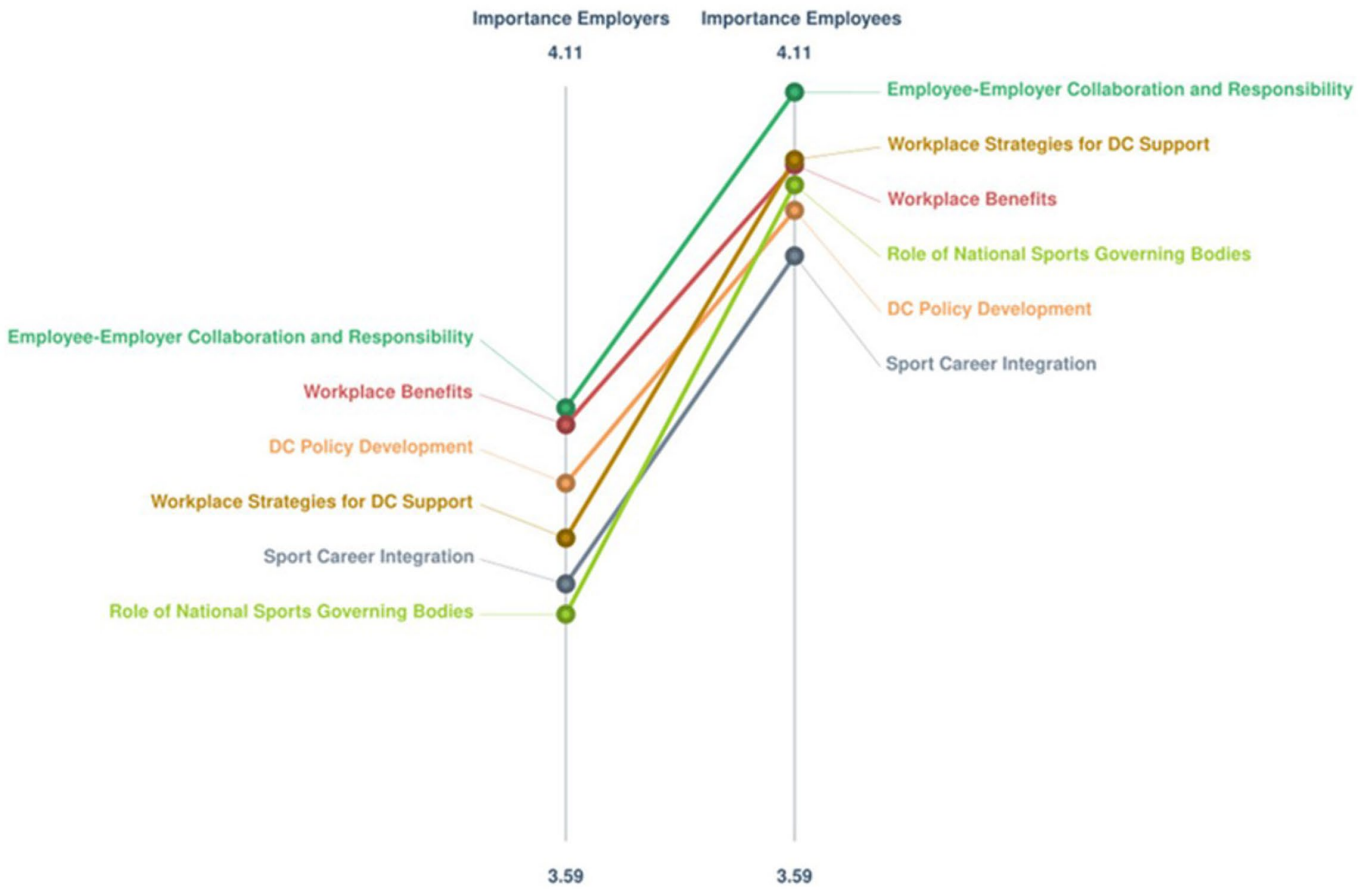
Employers-employees co-creation pattern match graph for the importance ratings; *Colored lines represents clusters: Red = Workplace benefits; Light green = Role of national sports governing bodies; Orange = Dual career policy development; Dark green = Employee-employer collaboration and responsibility; Grey = Sport career integration; Brown = Workplace strategies for dual career support.

**Figure 8:**
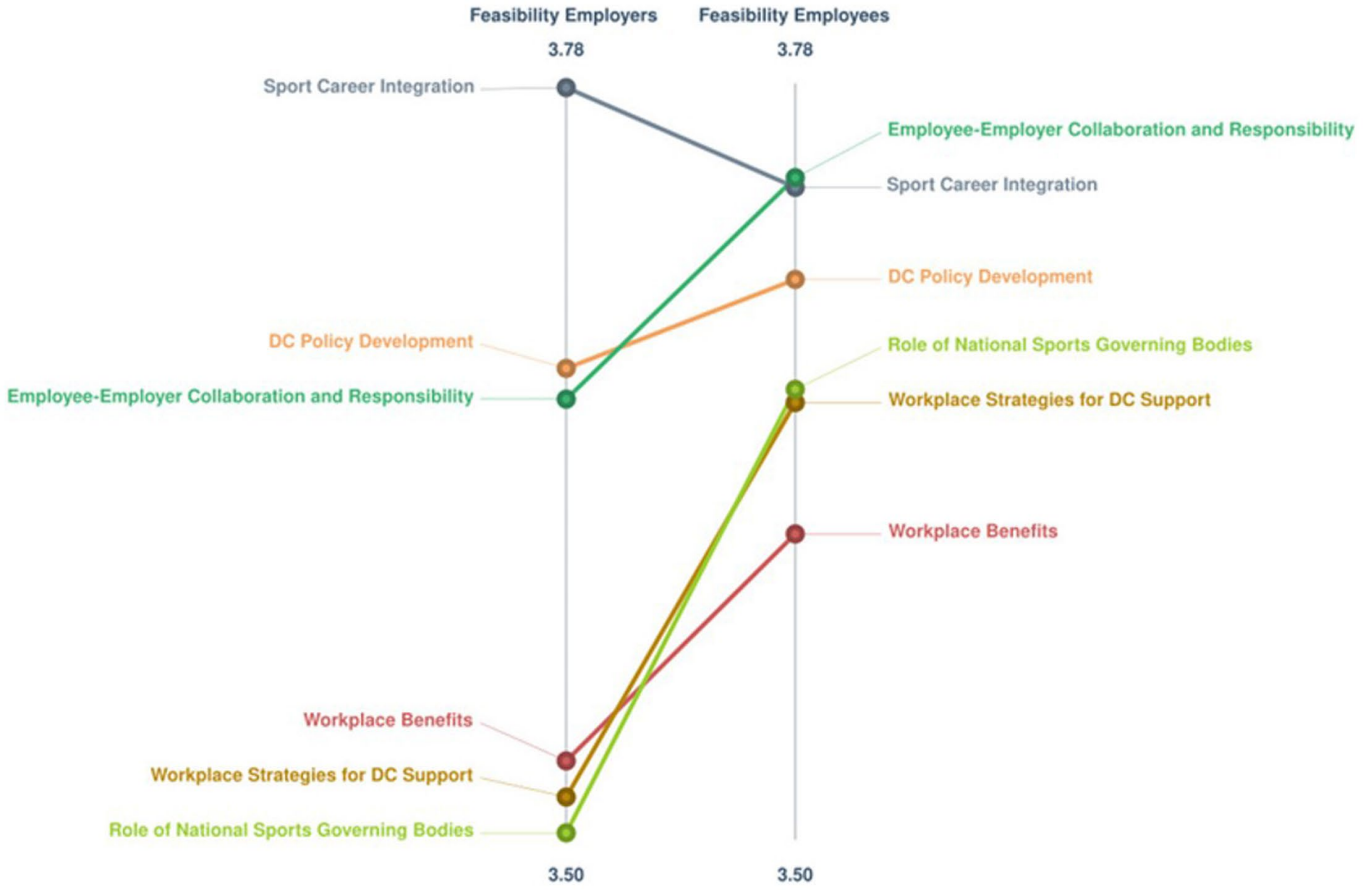
Employers-employees co-creation pattern match graph for the feasibility ratings; *Colored lines represents clusters: Red = Workplace benefits; Light green = Role of national sports governing bodies; Orange = Dual career policy development; Dark green = Employee-employer collaboration and responsibility; Grey = Sport career integration; Brown = Workplace strategies for dual career support.

When the Employers and Employees subgroups were considered together, the BRAVA-DC research team also agreed on a 6-cluster map, which reported a 0.14 stress index indicating a high degree of consistency (Figure 5). The supplementary Table 3 illustrates the six clusters with their corresponding statements and respective ratings. The findings indicate that one cluster (Sport Career Integration, 6 statements) related to the micro (i.e., the employee) DC dimension, one cluster (Employee-Employer Collaboration and Responsibility, 4 statements) related to the meso (i.e., interpersonal relationships) DC dimension, one cluster (Workplace Strategies for DC Support, 19 statements) related to the macro (i.e., working environment) DC dimension, two clusters related to the organizational (Workplace Benefits, 4 statements; and Dual Career Policy Development, 5 statements) DC dimension, and one cluster (Role of National Sports Governing Bodies, 12 statements) related to the policy dimension, respectively. For importance, only one cluster (DC Policy Development) reported a mean score < 4.0-point (3.7 ± 0.2-point) even though it reported the highest score for feasibility (4.0 ± 0.1-point). Figure 6 illustrates the Go-Zone, with an *r* = 0.62, resulting the higher alignment between the x and y variables with respect to that of the two subgroups. The statements were mainly distributed to clusters quadrant IV (*n* = 19) and quadrant II (17), with the statement 13 reporting the highest value for importance and statements 20 and 25 the highest value for feasibility, whereas the statement 34 showed the lowest value for importance and the statement 23 the lowest value for feasibility. For the importance and feasibility within the Co-Creation Scenario, the Tables 1, 2 report the comparisons between the Employers and Employees relative to the ratings of importance and feasibility, whereas the Figures 7, 8 show the pattern matches of the clusters. Differences (*p* < 0.05) emerged for both importance (clusters number 2, 4, and 6), and feasibility (clusters number 2, and 6), with highest scores for the employee-sportspersons. Figure 6 illustrates a go-zone graph representing all 50 statements. The Pearson Product Moment Correlation coefficient was *r* = 0.62. Quadrant IV (green), located in the top right, includes 19 statements rated above the average concerning both high im-portance and high feasibility.

**Table 1:** Employers and employees co-creation scenario importance ratings comparisons.

	Importance ratings							
	Employers (*N* = 37)		Employees (*N* = 143)		*p*-value	*t*-value	DF	d ES
Cluster number and name	Average	Variance	Average	Variance				
Workplace benefits	3.88	0.04	4.06	0.03	0.219	1.374	6	0.97
Role of national sports governing bodies	3.75	0.03	4.04	0.01	<0.001	5.148	22	2.10
DC policy development	3.84	0.06	4.03	0.01	0.133	1.671	8	1.06
Employee-employer collaboration and responsibility	3.89	0.01	4.11	0.01	0.021	3.092	6	2.19
Sport career integration	3.77	0.06	3.99	0.02	0.079	1.960	10	1.13
Workplace strategies for DC support	3.80	0.05	4.06	0.04	<0.001	3.834	36	1.27

DF, degrees of freedom; d ES, Cohen’s d; DC, Dual career.

**Table 2:** Employers and employees co-creation scenario feasibility ratings comparisons.

	Feasibility ratings							
	Employers (*N* = 37)		Employees (*N* = 143)		*p*-value	*t*-value	DF	d ES
Cluster number and name	Average	Variance	Average	Variance				
Workplace benefits	3.53	0.06	3.61	0.04	0.621	0.521	6	0.37
Role of national sports governing bodies	3.50	0.02	3.66	0.02	0.013	2.706	22	1.16
DC policy development	3.67	0.04	3.70	0.03	0.791	0.274	8	0.17
Employee-employer collaboration and responsibility	3.66	0.01	3.74	0.01	0.346	1.024	6	0.72
Sport career integration	3.77	0.04	3.74	0.03	0.739	0.343	10	0.20
Workplace strategies for DC support	3.51	0.05	3.66	0.02	0.020	2.429	36	0.79

DF, degrees of freedom; d ES, Cohen’s d; DC, Dual career.

## 4 Discussion

In capitalizing the combined knowledge, experience, and viewpoints of a broad representation of European managers and employee-sportspersons, the BRAVA-DC collaborative partnership offers a novel empirical approach to define a European framework of DC at the workplace, which could be valuable to guide the future DC research agenda and subsequent translation into policies (European Commission, 2012; Capranica and Guidotti, 2016; Capranica et al., 2021). Furthermore, the separate and combined analyses of the needs and visions of managers and employee-sportspersons allow the identification of specific aspects, which might help viable development of DC at the workplace. The present findings suggest priorities for changes at the DC micro, meso, macro and policy levels, and envisage flexible models for aligning brands values through DC of the employee-sportspersons.

Despite in the last decade the EU Guidelines succeeded in raising the awareness on the necessity to implement policies and research on possible actions for allowing the combination of sports and education commitments of the sportspersons, at present business-oriented companies do not offer flexible working conditions and arrangements enabling a sportspersons to prepare for and compete in athletic events as recommended by the European DC guidelines (European Commission, 2012, 2020b; Capranica and Guidotti, 2016; Stambulova and Wylleman, 2019; Moreno et al., 2021; Sina et al., 2021). In considering that companies are recognized social obligations in interacting with diverse community of stakeholders, they could establish an effective synergy between their vision (i.e., internal values) and their image (i.e., external dimension) through a proactive translation of DC into their policies for facilitating the employment of sportspersons and ameliorating their working conditions for future working career progression (Hatch and Majken, 2008; Balmer, 2012; Mingione, 2015; Yang et al., 2020; Hong and Fraser, 2023). In providing their opinion, in this study the managers scored the 50 DC statements more important (mean range: 3.7–3.9-point) than feasible (mean range: 3.5–3.7-point), clustering the majority of them (84%) in relation to the macro and policy DC dimensions (i.e., working environment and organization, and the national/international policies) and including most of them (81%) in the Quadrant IV. These findings highlight that managers deem relevant an innovative approach to brand-related DC vision, services, processes, and strategies, which could be aligned to national and European policies and ensure consistency and coherency between internal and external elements of the corporate brand (Mingione, 2023). Furthermore, the three statements pertaining the DC micro (i.e., the sportspersons) and meso (i.e., the relationship between the sportspersons and their managers) dimensions included in the Quadrant IV (i.e., numbers 22, 40, and 38) underline an employee-centered micro-CSR internal corporate practices, which could influence the DC employees’ affiliation, esteem of the corporation, and job satisfaction (Harrison and Wicks, 2013; Low and Siegel, 2019). In fact, the company could boost the employee-sportspersons’ performance, their attachment and involvement to the everyday activities and organization through aligning the brand values and DC practices (Markovic et al., 2018; Carlini and Grace, 2021). Furthermore, sportspersons have been considered valuable human resources for the business sector and could have an activist potential for the implementation of DC in CSR policies, specific pro-social behaviors, or CSR-related internal and external company communication (Moreno et al., 2021; Girschik et al., 2022). The managers recognized also the difficulty to implement seven important DC aspects (Quadrant I), which are mainly related to national/international bodies providing educational services and financial support for companies, as well as responsibilities for allowing an effective combination of sports and working commitments. Actually, the efforts and guidelines of the European Parliament and the European Commission contributed substantially to the recognition of the needs of student-sportsperson and the implementation of flexible DC models at the academic level, so that it is possible that in a near future the European DC discourse will foster also an evolution of DC at the workplace (Guidotti et al., 2015; Capranica and Guidotti, 2016; Stambulova and Wylleman, 2019). Finally, companies should implement their cooperation also with National organizations and sport governing bodies, which could leverage resources and expertise to promote CSR programs and initiatives addressing employment opportunities, labor relations, and well-established support networks that effectively help the sportspersons coping with different stressors and achieving their goals through work and sports commitments (Hong and Fraser, 2023).

While during the last decade the integration of sport and academic career has been conceived during the developmental years of a sportsperson, at present it is still difficult to envisage the combination of sports and work, even though sportspersons express values and competences considered empowering human resources for the business sector in guaranteeing goal achievements and success in dealing with work demands. In fact, recent empirical insights uncovered the sportspersons perceptions of lack of encouragement, adversities and barriers in their working environment and labor relations, which impact on their psychological wellbeing (EU Athletes, 2016; Robbins et al., 2019, 2020; Moreno et al., 2021; Hong and Fraser, 2023; Mingione et al., 2024). Over the last decades, companies have incorporated the employee wellness programs to foster a healthy and productive workforce (Stefaniec et al., 2022). The employee-sportspersons have expectations on improved sport-work balance and emotional or financial rewards, which could help them progressing along their career pathways, managing the sport- and work-related stress and preventing burnouts, and accommodate their social and family relationships and support (Moreno et al., 2021; Marshall et al., 2022). In the present study, the employee concept map could help us understanding the employee-sportspersons’ vision, which could boost DC resources and processes promoting engagement practices for inclusion through co-creative CSR activities (Rupp et al., 2006; Rupp and Mallory, 2015). With respect to their employer counterparts, the employee-sportspersons confirmed their high needs to implement the DC at the workplace by attributing higher values to the importance (mean range: 4.0–4.1-point) of the 50 DC statements yet recognizing similar implementation difficulties (feasibility mean range: 3.6–3.7-point). At the personal (i.e., micro), the relationship with employers (i.e., meso), and the working environment (i.e., macro) DC dimensions, sportspersons value especially relevant and feasible (Quadrant IV) to establish a regular and transparent communication to raise the awareness of the DC needs to improve the alignment of CSR policies and support, as well as to identify potential added value to generate positive external authentic brand reputation (Mingione and Leoni, 2020; Mingione, 2023; Mingione et al., 2024). In line with the overall lack of efficient institutionalized support for achieving a better work-sport balance reported in the literature (Moreno et al., 2021), employee-sportspersons attributed a special role to the national/international policies, which are expected to offer DC guidance and support, and to establish effective DC networks including academic institutions, sport bodies, and authorities to develop synergies for the implementation of innovative DC practices. In this framework, recent ERASMUS+Sport Collaborative partnerships focusing on employee-sportspersons could contribute to raise the awareness for accommodating the DC challenges at the workplace (Capranica et al., 2021; Mittag et al., 2022). The five statements deemed important but considered less implementable (i.e., Quadrant I) relate to the financial support, availability of paid sports leaves to attend training camps and competitions, and flexible working conditions allowing proper rest and healthy lifestyles. In considering that working from home and flexible working schedules have been widely adopted during the COVID-19 pandemic, it could be possible that employers consider distance working a viable option with positive impact on the sportsperson productivity (Maon et al., 2021; Stefaniec et al., 2022). To guarantee the employment, the salary, and the social security of elite Italian employee-paralympic athletes of the public or the private sectors who need a sport-leave within the limits of 90 days per year and a maximum of 30 continuous days, the Italian Paralympic Committee adopted a good practice of reimbursing the respective employers who request it, within the limits of 1 million euros per year starting from 2024 (Comitato Paralimpico Italiano, 2023).

A tight coupling between the employers and the employees proved to be beneficial both for the companies and their employees, with human resource departments urged to co-develop and implement programs focused on diversity, wellness, or work-life balance of their employees (European Commission, 2011, 2017, 2019, 2022a; Gond et al., 2011, 2017; App et al., 2012; Stefaniec et al., 2022). In the present study, the combination of the employers’ and employee-sportspersons’ opinions generate a co-creation scenario of DC at the workplace, which confirmed the high interest in the alignment of brand values through DC (cluster mean: 4.0-point) and sheds light on how its growth and structure could be implemented (cluster mean: 3.6-point). All the 6 clusters are represented by the 19 statements showing the highest values to the importance and feasibility (Quadrant IV), highlighting that all the DC dimensions should be considered, from the individuals and their relationships to the company’s vision and organization, and the society at large. Expectations at the micro and meso DC levels pertain a deep understanding of the sportspersons’ and company’s needs to be complemented by a clear and constant communication between the employer and the employee to create values, social, and for-profit congruence, whereas at the company’s organizational level a supportive network with other stakeholders (i.e., sports organizations, sports industries, educational institutions, governmental bodies) is envisaged to facilitate the employment of sportspersons based on the recognition of their experiences, competences and skills, and the definition of contracts tailoring duties, schedules, arrangements to make the best out of their commitments and prospective career progressions. Furthermore, national/international governing bodies are requested to establish a dedicated DC office to promote companies supporting DC, to provide financial incentives for the workplace to support paid sports leaves, and information on DC employment opportunities, thus contributing to the construction of a cultural discourse and the structuring of employee DC programs. In referring to a multi-level stakeholder networks perspective including employees, employers, and policy makers at sports organizations and governmental levels, this scenario generated a concept map that subsumes the limitations of a single level dyadic approach to co-create the alignment of brand and sports values through the DC at the workplace (Michela and Russell, 2022; Ruvalcaba et al., 2022; Mingione, 2023).

In developing a deep understanding of DC organizational-level CSR-related processes and potential bottom-up inspired changes related to a brand’s CSR strategy, and in disseminating best practices for the benefits of the company and employee-sportspersons, also a multi-level research design could have a relevant role for the advancement of a European DC discourse (Capranica and Guidotti, 2016; Akhouri and Chaudhary, 2019; Stambulova and Wylleman, 2019). Thus, future studies are needed to provide structured data collection on the effects of networks, synergies, and interactions between different stakeholders in the field of brand value alignment though DC.

## 5 Conclusion

The involvement of employers and employee-sportspersons in a concept mapping research design focused on DC at the workplace allowed to go beyond the dyadic employer-employee relationship and presented opportunities for an involvement of multiple stakeholders in value creation. The understanding of the roles and positions each stakeholder holds in the network and their influence in shaping value creation activities is vital to advance the European DC discourse. While the implementation of the corporate internal congruence could be achieved in focusing on the creation of structural links and cooperation between stakeholders at micro and meso DC levels, the corporate external congruence could be promoted by the creation of structural collaborations at macro and policy levels that respond to the societal quest of sustainability through the adoption of sport as a driven for human resource role in CSR brand values connected to the valorization of the sport community. Overall, the present findings can help companies planning alignment strategies to consider the different actors of the DC stakeholder networks.

## Data Availability

The raw data supporting the conclusions of this article will be made available by the authors, without undue reservation.

## Footnotes

^1^https://olympics.com/athlete365/topics/career-plus

^2^https://groupwisdom.com
