# Supplementary material for: Dual career in the workplace: co-creation of a conceptual framework by employers and employee-sportspersons incorporating corporate social responsibility and brand alignment
Source: Front Psychol. 2024 Sep 25;15:1432850. doi: 10.3389/fpsyg.2024.1432850 (PMC11461304; doi:10.3389/fpsyg.2024.1432850)
Supplement: Supplementary file 1 [file Table_1.DOCX]

Supplementary Material

**Supplementary table 1.** Employer statements, average ratings and Go-zone quadrant.

|  | | Mean rating ^a^ | |  |
| --- | --- | --- | --- | --- |
| Cluster name and statement – Employer | | Importance | Feasibility | Go-zone ^b^ |
| N | **Cluster 1: Financial Resources for Dual Career (DC)** | **3.9±0.2** | **3.6±0.2** |  |
| 1 | The Workplace, National Governing Bodies, and Educational Institutions to create a network of apprenticeships, internships and placements so that sportspersons (e.g., athletes, coaches, physical trainer, sports manager) can gain DC experience | 4.03 | 3.8 | IV |
| 4 | To identify and reward the workplace that specifically offer DC sportspersons (e.g., athletes/coaches/physical trainer/sports manager) employment and support during and/or after sport career | 3.81 | 3.5 | II |
| 18 | To implement appropriate financial support structures to support and facilitate the DC employee commitments | 3.73 | 3.3 | I |
|  |  |  |  |  |
|  | **Cluster 2: National/International Bodies** | **3.7±0.2** | **3.5±0.2** |  |
| 5 | National policies should foster the development/implementation of DC at workplace, brand alignment and Corporate Social Responsibility (CSR) | 3.9 | 3.6 | IV |
| 8 | A dedicated national/international office should support DC by providing entrepreneurship training to DC employees | 3.8 | 3.5 | II |
| 9 | A dedicated national/international office should support DC by providing quality certification for companies supporting DC (i.e. a national/international quality mark) | 3.5 | 3.5 | II |
| 11 | A dedicated national/international office should support DC by providing managerial training for successfully managing DC employees | 3.8 | 3.6 | IV |
| 12 | To introduce/implement national economic incentives for the workplace to support DC employees (e.g., co-fund DC salary, DC employment quotas etc.) | 3.4 | 3.1 | II |
| 15 | European policies should foster the development/implementation of DC at workplace, brand alignment and CSR | 3.6 | 3.7 | III |
| 19 | A dedicated national/international office should support DC by providing promotion of companies supporting DC | 3.9 | 3.7 | IV |
| 26 | A dedicated national/international office should support DC by providing Information on DC and DC employment opportunities | 3.9 | 3.5 | I |
| 28 | To develop and implement a nationwide promotional campaign regarding DC and its advantages to society and the workplace | 3.8 | 3.8 | III |
| 29 | National Governing Bodies (NGBs)/Institutions of sport should prepare portfolios on DC cases in companies and share with relevant stakeholders (e.g., workplace managers, DC employees, sport-related institutions etc.) | 3.6 | 3.6 | III |
| 35 | A dedicated national/international office should support DC by providing grants/scholarships for DC employees' quality of life (e.g., paid leave during sport competitions / training / injuries / restricted performance) | 3.9 | 3.3 | I |
| 37 | NGBs of sport should provide planning and workplace training for DC employers to meet DC employee needs | 3.8 | 3.6 | IV |
| 41 | NGBs/Institutions of sport should provide guidance and support to the workplace for different types of sponsorship that can be gained by DC employees | 3.5 | 3.6 | II |
| 43 | NBBs of sport should provide planning, workplace and coping training for DC employees to meet employer needs | 3.8 | 3.5 | I |
| 50 | NGBs of sport illustrate to the workplace the potential value of DC employees to their organisation and how to align with corporate brand and CSR policies | 3.8 | 3.5 | I |
|  |  |  |  |  |
|  | **Cluster 3: Employee Responsibilities** | **3.8±0.2** | **3.7±0.1** |  |
| 6 | Employees should establish a DC support network to share ideas, practices, arrangements, etc. | 3.7 | 3.8 | III |
| 22 | DC employees and their employers regularly (e.g., on a six-month basis) agree a work and sport commitment schedule | 3.9 | 3.8 | IV |
| 27 | DC employee (e.g., athletes/coaches/physical trainer/sports manager) and employer take joint responsibility for the effective integration of work and sport and for long-term employment prospects and conditions | 3.9 | 3.6 | I |
| 40 | Managers and employees should be aware of the DC employees' needs during and/or after their sport career | 4.0 | 3.8 | IV |
|  |  |  |  |  |
|  | **Cluster 4: Promotion of DC** | **3.8±0.3** | **3.7±0.3** |  |
| 7 | To outline at an early stage the nature of the expectations between a named sport sponsor and the DC employee | 3.5 | 3.7 | III |
| 10 | "Elite sportspersons" (e.g., athletes/coaches/physical trainer/sports manager) and "sportspersons" (e.g., athletes/coaches/physical trainer/sports manager) should be considered an official job title | 3.5 | 3.4 | II |
| 20 | Transparency and open communication are crucial for an authentic/aligned CSR approach to DC | 4.3 | 3.9 | IV |
| 21 | To recognise that it may be more challenging for some industries/workplaces to provide effective DC supports | 3.8 | 3.9 | III |
| 23 | The benefit of a workplace DC corporate brand alignment & CSR policy should be measurable for both the workplace & society | 3.6 | 3.1 | II |
| 25 | To develop and promote DC employment opportunities within the sport related industries | 4.3 | 3.9 | IV |
| 30 | To recognise DC commitment and success as part of national sporting awards | 3.8 | 4.0 | III |
|  |  |  |  |  |
|  | **Cluster 5: Workplace/Employers** | **3.8±0.2** | **3.5±0.2** |  |
| 2 | The workplace should recognise that the absence of employers understanding of the DC challenges can directly affect the holistic health of the DC employee and their work-life balance | 3.8 | 3.4 | I |
| 13 | The Workplace should support DC employees through specific contracts/working arrangements (e.g., flexible working hours/part-time job/job sharing/ substitution/employment exchange to attend competitions and training camps) | 4.3 | 3.8 | IV |
| 14 | The Workplace should recognise value of DC employee to organisation and thus provide benefits to facilitate DC employee sport commitment (e.g., sponsorship, sport equipment, scholarships, transport etc.) | 3.8 | 3.7 | III |
| 16 | The Workplace should benchmark against organisations which demonstrate good employee DC support structures and practice | 3.7 | 3.4 | II |
| 17 | The Workplace should provide salary structures and levels and paid and unpaid leave that support and align with the DC employee part-time/flexible working needs | 3.8 | 3.5 | II |
| 24 | The Workplace should identify and recognise DC employees' skills/experience/competences as potential added value to generate external positive outcomes (e.g., CSR and brand ambassadors, brand reputation, potential collaboration with sporting companies, sport sponsorship) | 4.1 | 3.8 | IV |
| 31 | The workplace should recognise and accommodates the workplace challenges of DC employees, related to their specific needs, such as rest, recovery and proper nutrition and work commitments | 3.8 | 3.2 | II |
| 32 | The Workplace should support DC employees (e.g., athletes/coaches/physical trainer/sports manager) competing at any sport federation level, by focusing on their overall wellbeing and not only on return of investment | 3.8 | 3.2 | I |
| 34 | Some workplaces demonstrate their support, brand alignment and CSR to sport via sponsorship of athletes, sport organisations and events as expectations beyond this level (e.g., grants, recovery time) of support may not be realistic | 3.4 | 3.5 | II |
| 36 | To implement workplace support structures that recognise and facilitate the conflicting time demands between work and sport and the complex logistics of commitment to both careers (e.g., training and work locations, short notice for training camps etc.) | 3.7 | 3.3 | II |
| 39 | The Workplace should invest in the local community to support DC employees | 3.7 | 3.5 | II |
| 42 | The Workplace should provide DC mentoring for both managers and employees | 3.5 | 3.5 | II |
| 44 | The Workplace should support DC by recognising the co-created value stemming from the alignment between corporate brand strategies and CSR policies | 3.6 | 3.3 | II |
| 45 | The Workplace should verify/implement at the earliest stage possible mutual DC arrangements and commitments, which best meet both employer and employee’s needs (e.g., employer expectations, task oriented working outcomes and deadlines, training camps/competition dates etc.) | 4.1 | 3.8 | IV |
| 46 | That the workplace recognise that work commitment and career progression can be planned in parallel with sport commitments and progression | 4.2 | 3.6 | IV |
| 48 | The Workplace should recognise the DC employees' skills/experience/competences as potential value to generate internal positive outcomes (e.g., experience enhancement, exchange of ideas, and shared values such as goal orientation, competitiveness, perseverance, commitment to hard work and ethical-related issues) | 3.9 | 3.6 | IV |
| 49 | The Workplace should implement regular and effective communication between DC employee and employer regarding working and sport commitments | 4.0 | 3.9 | IV |
|  |  |  |  |  |
|  | **Cluster 6: Employer-Employee Cooperation and Alignment** | **3.8±0.2** | **3.5±0.3** |  |
| 3 | Employers should not have lower expectations/standards of work because of DC employees' sport commitment | 3.7 | 3.5 | II |
| 33 | To successfully co-create value, the workplace should aim to align and balance the stakeholders' (e.g., workplace managers, DC employees, sport-related institutions etc.) expectations and responsibilities | 3.7 | 3.6 | II |
| 38 | DC should be considered in corporate strategies and CSR policies because it represents a tangible manifestation of ethical values and moral attitude, both typical of a conscious and purpose-approach pertaining to leading organisations | 4.1 | 3.7 | IV |
| 47 | To enhance existing workplace sport facilities and equipment to support DC employee’s sport commitment | 3.5 | 3.1 | II |

Notes: ^a^ Mean rating from 0 (least important/feasible) to 5 (most important/feasible); ^b^ Go-Zone quadrants: I, low importance/low feasibility; II, high importance/low feasibility; III, low importance/high feasibility; IV, high importance/high feasibility.

**Supplementary table 2.** Employee statements, average ratings and Go-zone quadrant

|  | | Mean rating ^a^ | |  |
| --- | --- | --- | --- | --- |
| Cluster name and statement – Employee | | Importance | Feasibility | Go-zone ^b^ |
| N | **Cluster 1: Workplace Support for Dual Careers** | **4.1±0.2** | **3.7±0.2** |  |
| 1 | The Workplace, National Governing Bodies, and Educational Institutions to create a network of apprenticeships, internships, and placements so that sportspersons (e.g., athletes, coaches, physical trainer, sports manager) can gain Dual Career experience | 4.3 | 3.8 | IV |
| 4 | To identify and reward the workplace that specifically offer Dual Career sportspersons (e.g., athletes/coaches/physical trainer/sports manager) employment and support during and/or after sport career | 4.2 | 3.8 | IV |
| 12 | To introduce/implement national economic incentives for the workplace to support Dual Career employees (e.g., co-fund Dual Career salary, Dual Career employment quotas etc.) | 4.0 | 3.5 | II |
| 47 | To enhance existing workplace sport facilities and equipment to support Dual Career employee’s sport commitments | 3.9 | 3.6 | II |
|  | **Cluster 2: National/International Support for Dual Career** | **4.0±0.1** | **3.7±0.1** |  |
| 5 | National policies should foster the development/implementation of Dual Career at workplace, brand alignment and Corporate Social Responsibility | 4.1 | 3.5 | I |
| 8 | A dedicated national/international office should support Dual Career by providing entrepreneurship training to Dual Career employees | 4.0 | 3.5 | II |
| 9 | A dedicated national/international office should support Dual Career by providing quality certification for companies supporting Dual Career (i.e. a national / international quality mark) | 4.0 | 3.7 | III |
| 11 | A dedicated national/international office should support Dual Career by providing managerial training for successfully managing Dual Career employees | 4.0 | 3.7 | II |
| 15 | European policies should foster the development/implementation of Dual Career at workplace, brand alignment and Corporate Social Responsibility | 4.0 | 3.6 | II |
| 19 | A dedicated national/international office should support Dual Career by providing promotion of companies supporting Dual Career | 4.0 | 3.8 | III |
| 26 | A dedicated national/international office should support Dual Career by providing Information on Dual Career and Dual Career employment opportunities | 4.2 | 4.0 | IV |
| 29 | National Governing Bodies/Institutions of sport should prepare portfolios on Dual Career cases in companies and share with relevant stakeholders (e.g., workplace managers, Dual Career employees, sport-related institutions etc.) | 3.8 | 3.5 | II |
| 35 | A dedicated national/international office should support Dual Career by providing grants/scholarships for Dual Career employees' quality of life (e.g., paid leave during sport competitions / training / injuries / restricted performance) | 4.3 | 3.8 | IV |
| 37 | National Governing Bodies of sport should provide planning and workplace training for Dual Career employers to meet Dual Career employee needs | 4.0 | 3.6 | II |
| 41 | National Governing Bodies /Institutions of sport should provide guidance and support to the workplace for different types of sponsorship that can be gained by Dual Career employees | 4.1 | 3.7 | IV |
| 43 | National Governing Bodies of sport should provide planning, workplace and coping training for Dual Career employees to meet employer needs | 4.0 | 3.7 | III |
| 50 | National Governing Bodies of sport illustrate to the workplace the potential value of Dual Career employees to their organisation and how to align with corporate brand and Corporate Social Responsibility policies | 3.9 | 3.6 | II |
|  | **Cluster 3: Dual Career Policy Development** | **4.0±0.1** | **3.6±0.3** |  |
| 18 | To implement appropriate financial support structures to support and facilitate the Dual Career employee commitments | 4.1 | 3.5 | I |
| 23 | The benefit of a workplace Dual Career corporate brand alignment and Corporate Social Responsibility policy should be measurable for both the workplace and society | 3.9 | 3.4 | II |
| 25 | To develop and promote Dual Career employment opportunities within the sport related industries | 4.1 | 4.1 | IV |
| 28 | To develop and implement a nationwide promotional campaign regarding Dual Career and its advantages to society and the workplace | 3.9 | 3.7 | III |
| 38 | Dual Career should be considered in corporate strategies and Corporate Social Responsibility policies because it represents a tangible manifestation of ethical values and moral attitude, both typical of a conscious and purpose-approach pertaining to leading organisations | 3.9 | 3.4 | II |
|  | **Cluster 4: Employer and Employee Obligations** | **4.0±0.1** | **3.7±0.1** |  |
| 3 | Employers should not have lower expectations/standards of work because of Dual Career employees' sport commitment | 4.0 | 3.6 | II |
| 22 | Dual Career employees and their employers regularly (e.g., on a six-month basis) agree a work and sport commitment schedule | 4.1 | 3.9 | IV |
| 27 | Dual Career employee (e.g., athletes/coaches/physical trainer/sports manager) and employer take joint responsibility for the effective integration of work and sport and for long-term employment prospects and conditions | 4.1 | 3.7 | IV |
| 40 | Managers and employees should be aware of the Dual Career employees' needs during and/or after their sport career | 4.2 | 3.8 | IV |
|  | **Cluster 5: Dual Career Initiative and Recognition** | **4.0±0.2** | **3.7±0.2** |  |
| 6 | Employees should establish a Dual Career support network to share ideas, practices, arrangements, etc | 3.9 | 3.8 | III |
| 7 | To outline at an early stage the nature of the expectations between a named sport sponsor and the Dual Career employee | 3.9 | 3.8 | III |
| 10 | "Elite sportspersons" (e.g., athletes/coaches/physical trainer/sports manager) and "sportspersons" (e.g., athletes/coaches/physical trainer/sports manager) should be considered an official job title | 3.9 | 3.5 | II |
| 20 | Transparency and open communication are crucial for an authentic/aligned Corporate Social Responsibility approach to Dual Career | 4.3 | 4.1 | IV |
| 21 | To recognise that it may be more challenging for some industries/workplaces to provide effective Dual Career supports | 4.1 | 3.8 | IV |
| 30 | To recognise Dual Career commitment and success as part of national sporting awards | 3.9 | 3.6 | II |
|  | **Cluster 6: Employer Support for Dual Careers** | **4.1±0.2** | **3.6±0.1** |  |
| 2 | The workplace should recognise that the absence of employers understanding of the Dual Career challenges can directly affect the holistic health of the Dual Career employee and their work-life balance | 4.2 | 3.8 | IV |
| 13 | The Workplace should support Dual Career employees through specific contracts/working arrangements (e.g., flexible working hours/part-time job/job sharing/ substitution/employment exchange to attend competitions and training camps) | 4.4 | 3.9 | IV |
| 14 | The Workplace should recognise value of Dual Career employee to organisation and thus provide benefits to facilitate Dual Career employee sport commitment (e.g., sponsorship, sport equipment, scholarships, transport etc.) | 4.1 | 3.7 | IV |
| 16 | The Workplace should benchmark against organisations which demonstrate good employee Dual Career support structures and practice | 3.8 | 3.6 | II |
| 17 | The Workplace should provide salary structures and levels and paid and unpaid leave that support and align with the Dual Career employee part-time/flexible working needs | 4.2 | 3.6 | I |
| 24 | The Workplace should identify and recognise Dual Career employees' skills/experience/competences as potential added value to generate external positive outcomes (e.g., Corporate Social Responsibility and brand ambassadors, brand reputation, potential collaboration with sporting companies, sport sponsorship) | 4.2 | 3.8 | IV |
| 31 | The workplace should recognise and accommodates the workplace challenges of Dual Career employees, related to their specific needs, such as rest, recovery and proper nutrition and work commitments | 4.1 | 3.5 | I |
| 32 | The Workplace should support Dual Career employees (e.g., athletes/coaches/physical trainer/sports manager) competing at any sport federation level, by focusing on their overall wellbeing and not only on return of investment | 4.0 | 3.5 | II |
| 33 | To successfully co-create value, the workplace should aim to align and balance the stakeholders' (e.g., workplace managers, Dual Career employees, sport-related institutions etc.) expectations and responsibilities | 4.0 | 3.6 | II |
| 34 | Some workplaces demonstrate their support, brand alignment and Corporate Social Responsibility to sport via sponsorship of athletes, sport organisations and events as expectations beyond this level (e.g., grants, recovery time) of support may not be realistic | 3.7 | 3. 5 | II |
| 36 | To implement workplace support structures that recognise and facilitate the conflicting time demands between work and sport and the complex logistics of commitment to both careers (e.g., training and work locations, short notice for training camps etc.) | 4.1 | 3.5 | I |
| 39 | The Workplace should invest in the local community to support Dual Career employees | 3.9 | 3.6 | II |
| 42 | The Workplace should provide Dual Career mentoring for both managers and employees | 3.8 | 3.6 | II |
| 44 | The Workplace should support Dual Career by recognising the co-created value stemming from the alignment between corporate brand strategies and Corporate Social Responsibility policies | 3.8 | 3.6 | II |
| 45 | The Workplace should verify/implement at the earliest stage possible mutual Dual Career arrangements and commitments, which best meet both employer and employee’s needs (e.g., employer expectations, task oriented working outcomes and deadlines, training camps/competition dates etc.) | 4.1 | 3.8 | IV |
| 46 | That the workplace recognise that work commitment and career progression can be planned in parallel with sport commitments and progression | 4.3 | 3.8 | IV |
| 48 | The Workplace should recognise the Dual Career employees' skills/experience/competences as potential value to generate internal positive outcomes (e.g., experience enhancement, exchange of ideas, and shared values such as goal orientation, competitiveness, perseverance, commitment to hard work and ethical-related issues) | 4.1 | 3.8 | IV |
| 49 | The Workplace should implement regular and effective communication between Dual Career employee and employer regarding working and sport commitments | 4.3 | 4.0 | IV |

Notes: ^a^ Mean rating from 0 (least important/feasible) to 5 (most important/feasible); ^b^ Go-Zone quadrants: I, low importance/low feasibility; II, high importance/low feasibility; III, low importance/high feasibility; IV, high importance/high feasibility.

**Supplementary table 3.** Co-creation statements, average ratings and Go-zone quadrant.

|  | | Mean rating ^a^ | | | |  | |
| --- | --- | --- | --- | --- | --- | --- | --- |
| Cluster name and statement – co-creation | | Importance | | Feasibility | | Go-zone^b^ | |
| N | **Cluster 1: Workplace Benefits** | | **4.0±0.2** | | **3.6±0.2** | |  |
| 1 | The Workplace, National Governing Bodies, and Educational Institutions to create a network of apprenticeships, internships and placements so that sportspersons (e.g., athletes, coaches, physical trainer, sports manager) can gain Dual Career experience | | 4.2 | | 3.8 | | IV |
| 4 | To identify and reward the workplace that specifically offer Dual Career sportspersons (e.g., athletes/coaches/physical trainer/sports manager) employment and support during and/or after sport career | | 4.1 | | 3.7 | | IV |
| 23 | The benefit of a workplace Dual Career corporate brand alignment and Corporate Social Responsibility policy should be measurable for both the workplace and society | | 3.8 | | 3.4 | | II |
| 38 | Dual Career should be considered in corporate strategies and Corporate Social Responsibility policies because it represents a tangible manifestation of ethical values and moral attitude, both typical of a conscious and purpose-approach pertaining to leading organisations | | 3.9 | | 3.5 | | II |
|  | **Cluster 2: Role of National Sports Governing Bodies** | | **4.0±0.1** | | **3.6±0.1** | |  |
| 5 | National policies should foster the development/implementation of Dual Career at workplace, brand alignment and Corporate Social Responsibility | | 4.1 | | 3.5 | | I |
| 8 | A dedicated national/international office should support Dual Career by providing entrepreneurship training to Dual Career employees | | 3.9 | | 3.5 | | II |
| 9 | A dedicated national/international office should support Dual Career by providing quality certification for companies supporting Dual Career (i.e. a national / international quality mark) | | 3.9 | | 3.7 | | III |
| 12 | To introduce/implement national economic incentives for the workplace to support Dual Career employees (e.g., co-fund Dual Career salary, Dual Career employment quotas etc.) | | 3.9 | | 3.4 | | II |
| 19 | A dedicated national/international office should support Dual Career by providing promotion of companies supporting Dual Career | | 4.0 | | 3.8 | | IV |
| 26 | A dedicated national/international office should support Dual Career by providing Information on Dual Career and Dual Career employment opportunities | | 4.1 | | 3.9 | | IV |
| 29 | National Governing Bodies/Institutions of sport should prepare portfolios on Dual Career cases in companies and share with relevant stakeholders (e.g., workplace managers, Dual Career employees, sport-related institutions etc.) | | 3.8 | | 3.5 | | II |
| 35 | A dedicated national/international office should support Dual Career by providing grants/scholarships for Dual Career employees' quality of life (e.g., paid leave during sport competitions / training / injuries / restricted performance) | | 4.2 | | 3.7 | | IV |
| 37 | National Governing Bodies of sport should provide planning and workplace training for Dual Career employers to meet Dual Career employee needs | | 4.0 | | 3.6 | | II |
| 41 | National Governing Bodies /Institutions of sport should provide guidance and support to the workplace for different types of sponsorship that can be gained by Dual Career employees | | 4.0 | | 3.7 | | III |
| 43 | National Governing Bodies of sport should provide planning, workplace and coping training for Dual Career employees to meet employer needs | | 4.0 | | 3.7 | | I |
| 50 | National Governing Bodies of sport illustrate to the workplace the potential value of Dual Career employees to their organisation and how to align with corporate brand and Corporate Social Responsibility policies | | 3.9 | | 3.6 | | II |
|  | **Cluster 3: DC Policy Development** | | **3.7±0.2** | | **4.0±0.1** | |  |
| 11 | A dedicated national/international office should support Dual Career by providing managerial training for successfully managing Dual Career employees | | 3.7 | | 4.0 | | III |
| 15 | European policies should foster the development/implementation of Dual Career at workplace, brand alignment and Corporate Social Responsibility | | 3.6 | | 3.9 | | II |
| 18 | To implement appropriate financial support structures to support and facilitate the Dual Career employee commitments | | 3.5 | | 4.0 | | I |
| 25 | To develop and promote Dual Career employment opportunities within the sport related industries | | 4.0 | | 4.1 | | IV |
| 28 | To develop and implement a nationwide promotional campaign regarding Dual Career and its advantages to society and the workplace | | 3.7 | | 3.9 | | III |
|  | **Cluster 4: Employee-Employer Collaboration and Responsibility** | | **4.1±0.1** | | **3.7±0.1** | |  |
| 3 | Employers should not have lower expectations/standards of work because of Dual Career employees' sport commitment | | 3.9 | | 3.6 | | I |
| 22 | Dual Career employees and their employers regularly (e.g., on a six-month basis) agree a work and sport commitment schedule | | 4.1 | | 3.8 | | IV |
| 27 | Dual Career employee (e.g., athletes/coaches/physical trainer/sports manager) and employer take joint responsibility for the effective integration of work and sport and for long-term employment prospects and conditions | | 4.1 | | 3.7 | | IV |
| 40 | Managers and employees should be aware of the Dual Career employees' needs during and/or after their sport career | | 4.2 | | 3.8 | | IV |
|  | **Cluster 5: Sport Career Integration** | | **4.0±0.2** | | **3.7±0.2** | |  |
| 6 | Employees should establish a Dual Career support network to share ideas, practices, arrangements, etc | | 3.8 | | 3.8 | | III |
| 7 | To outline at an early stage the nature of the expectations between a named sport sponsor and the Dual Career employee | | 3.8 | | 3.8 | | III |
| 10 | "Elite sportspersons" (e.g., athletes/coaches/physical trainer/sports manager) and "sportspersons" (e.g., athletes/coaches/physical trainer/sports manager) should be considered an official job title | | 3.9 | | 3.5 | | II |
| 20 | Transparency and open communication are crucial for an authentic/aligned Corporate Social Responsibility approach to Dual Career | | 4.3 | | 4.0 | | IV |
| 21 | To recognise that it may be more challenging for some industries/workplaces to provide effective Dual Career supports | | 4.0 | | 3.8 | | IV |
| 30 | To recognise Dual Career commitment and success as part of national sporting awards | | 3.9 | | 3.7 | | III |
|  | **Cluster 6: Workplace Strategies for DC Support** | | **4.0±0.2** | | **3.6±0.2** | |  |
| 2 | The workplace should recognise that the absence of employers understanding of the Dual Career challenges can directly affect the holistic health of the Dual Career employee and their work-life balance | | 4.2 | | 3.7 | | IV |
| 13 | The Workplace should support Dual Career employees through specific contracts/working arrangements (e.g., flexible working hours/part-time job/job sharing/ substitution/employment exchange to attend competitions and training camps) | | 4.4 | | 3.8 | | IV |
| 14 | The Workplace should recognise value of Dual Career employee to organisation and thus provide benefits to facilitate Dual Career employee sport commitment (e.g., sponsorship, sport equipment, scholarships, transport etc.) | | 4.0 | | 3.7 | | IV |
| 16 | The Workplace should benchmark against organisations which demonstrate good employee Dual Career support structures and practice | | 3.7 | | 3.6 | | II |
| 17 | The Workplace should provide salary structures and levels and paid and unpaid leave that support and align with the Dual Career employee part-time/flexible working needs | | 4.1 | | 3.6 | | I |
| 24 | The Workplace should identify and recognise Dual Career employees' skills/experience/competences as potential added value to generate external positive outcomes (e.g., Corporate Social Responsibility and brand ambassadors, brand reputation, potential collaboration with sporting companies, sport sponsorship) | | 4.2 | | 3. 8 | | IV |
| 31 | The workplace should recognise and accommodates the workplace challenges of Dual Career employees, related to their specific needs, such as rest, recovery and proper nutrition and work commitments | | 4.0 | | 3.5 | | I |
| 32 | The Workplace should support Dual Career employees (e.g., athletes/coaches/physical trainer/sports manager) competing at any sport federation level, by focusing on their overall wellbeing and not only on return of investment | | 4.0 | | 3.4 | | II |
| 33 | To successfully co-create value, the workplace should aim to align and balance the stakeholders' (e.g., workplace managers, Dual Career employees, sport-related institutions etc.) expectations and responsibilities | | 4.0 | | 3.6 | | II |
| 34 | Some workplaces demonstrate their support, brand alignment and Corporate Social Responsibility to sport via sponsorship of athletes, sport organisations and events as expectations beyond this level (e.g., grants, recovery time) of support may not be realistic | | 3.7 | | 3.5 | | II |
| 36 | To implement workplace support structures that recognise and facilitate the conflicting time demands between work and sport and the complex logistics of commitment to both careers (e.g., training and work locations, short notice for training camps etc.) | | 4.0 | | 3.5 | | I |
| 39 | The Workplace should invest in the local community to support Dual Career employees | | 3.8 | | 3.6 | | II |
| 42 | The Workplace should provide Dual Career mentoring for both managers and employees | | 3.8 | | 3.6 | | II |
| 44 | The Workplace should support Dual Career by recognising the co-created value stemming from the alignment between corporate brand strategies and Corporate Social Responsibility policies | | 3.8 | | 3.5 | | II |
| 45 | The Workplace should verify/implement at the earliest stage possible mutual Dual Career arrangements and commitments, which best meet both employer and employee’s needs (e.g., employer expectations, task oriented working outcomes and deadlines, training camps/competition dates etc.) | | 4.1 | | 3.8 | | IV |
| 46 | That the workplace recognise that work commitment and career progression can be planned in parallel with sport commitments and progression | | 4.3 | | 3.8 | | IV |
| 47 | To enhance existing workplace sport facilities and equipment to support Dual Career employee’s sport commitments | | 3.8 | | 3.5 | | II |
| 48 | The Workplace should recognise the Dual Career employees' skills/experience/competences as potential value to generate internal positive outcomes (e.g., experience enhancement, exchange of ideas, and shared values such as goal orientation, competitiveness, perseverance, commitment to hard work and ethical-related issues) | | 4.1 | | 3.7 | | IV |
| 49 | The Workplace should implement regular and effective communication between Dual Career employee and employer regarding working and sport commitments | | 4.2 | | 4.0 | | IV |

Note: ^a^ Mean rating from 0 (least important/feasible) to 5 (most important/feasible); ^b^ Go-Zone quadrants: I, low importance/low feasibility; II, high importance/low feasibility; III, low importance/high feasibility; IV, high importance/high feasibility.
